# MYC and KRAS cooperation: from historical challenges to therapeutic opportunities in cancer

**DOI:** 10.1038/s41392-024-01907-z

**Published:** 2024-08-21

**Authors:** Sílvia Casacuberta-Serra, Íñigo González-Larreategui, Daniel Capitán-Leo, Laura Soucek

**Affiliations:** 1 Peptomyc S.L., Barcelona, Spain; 2 Models of cancer therapies Laboratory, Vall d’Hebron Institute of Oncology, Cellex Centre, Hospital University Vall d’Hebron Campus, Barcelona, Spain; 3 Institució Catalana de Recerca i Estudis Avançats, Barcelona, Spain; 4 Department of Biochemistry and Molecular Biology, Universitat Autonoma de Barcelona, Bellaterra, Spain

**Keywords:** Oncogenes, Cancer therapy

## Abstract

RAS and MYC rank amongst the most commonly altered oncogenes in cancer, with RAS being the most frequently mutated and MYC the most amplified. The cooperative interplay between RAS and MYC constitutes a complex and multifaceted phenomenon, profoundly influencing tumor development. Together and individually, these two oncogenes regulate most, if not all, hallmarks of cancer, including cell death escape, replicative immortality, tumor-associated angiogenesis, cell invasion and metastasis, metabolic adaptation, and immune evasion. Due to their frequent alteration and role in tumorigenesis, MYC and RAS emerge as highly appealing targets in cancer therapy. However, due to their complex nature, both oncogenes have been long considered “undruggable” and, until recently, no drugs directly targeting them had reached the clinic. This review aims to shed light on their complex partnership, with special attention to their active collaboration in fostering an immunosuppressive milieu and driving immunotherapeutic resistance in cancer. Within this review, we also present an update on the different inhibitors targeting RAS and MYC currently undergoing clinical trials, along with their clinical outcomes and the different combination strategies being explored to overcome drug resistance. This recent clinical development suggests a paradigm shift in the long-standing belief of RAS and MYC “undruggability”, hinting at a new era in their therapeutic targeting.

## Introduction

Cancer is a complex and heterogeneous disease that stands as the second leading cause of death globally, claiming millions of lives every year. Contributing factors include an aging population, tobacco use, obesity, and environmental exposures. The leading cancer types worldwide are lung (12.4%), breast (11.6%), colorectum (9.6%), prostate (7.3%) and stomach (4.9%).^1^ The multifaceted nature of cancer presents unique challenges in diagnosis, treatment, and prevention, prompting a critical need to understand the intricate mechanisms driving its pathogenesis and therapeutic resistance.

Molecular oncology has revealed that mutations in specific regulatory genes are fundamental drivers of the disease. Among these alterations, dysregulation of key oncogenes and tumor suppressors plays a pivotal role in initiating and sustaining malignant transformation. Notably, RAS and MYC are among the most commonly deregulated oncogenes in cancer, with RAS mutations being the most frequent^2^ and MYC the most amplified.^3^ Given their widespread alteration across various cancer types and their significant role in tumorigenesis, both MYC and RAS have garnered considerable attention as promising therapeutic targets. However, their complex nature has long rendered them “undruggable”, and until recently, no drugs directly targeting them had reached clinical use.

RAS proteins are encoded by three ubiquitously expressed genes: KRAS, HRAS, and NRAS. Mutations in the RAS isoforms lead to constitutive activation of downstream signaling pathways involved in cell proliferation, survival, and invasion, fueling tumorigenesis. Of the RAS isoforms, KRAS is the most frequently mutated (85%), followed by NRAS (12%) and HRAS (3%).^4^ KRAS mutations are prevalent in approximately 20% of all human cancers, with particularly high frequencies observed in pancreatic ductal adenocarcinoma (PDAC; 88%), colorectal cancer (CRC; 50%), and non-small cell lung cancer (NSCLC; 32%).^5^ KRAS mutations typically manifest as activating mutations commonly occurring as single nucleotide substitutions, predominantly in four hotspot codons: 12, 13, 61, and 146. Among these, codon 12 exhibits the highest mutation frequency, accounting for nearly 90% of all KRAS mutations, with the G12D mutation being the most prevalent (29.19%), followed by G12V (22.97%) and G12C (13.43%).^6–8^ Notably, the specific activating alleles of KRAS vary considerably across different cancer types, suggesting potential variations in the signaling characteristics of the mutant proteins within distinct cellular environments. For instance, in PDAC and CRC, G12D and G12V are the most common alterations, whereas G12C showcases as the predominant mutant subtype in NSCLC. The occurrence of mutations in alternative codons varies, yet mutations outside of codon 12 constitute a notable portion of KRAS activating alleles in certain cancers. For instance, mutations in codons 13, 61, 117, and 146 are prevalent in CRC compared to NSCLC and PDAC. Interestingly, CRC distinguishes itself with its diverse array of alleles among the “big 3” cancers known for their high prevalence of KRAS mutations.^6^

KRAS mutations are also present in rare cancers, revealing a general mutation rate of 8.7%. Among these, G12D, G12V and G13D are identified as the most frequent mutations.^9^ In this context, the different oncogenic KRAS alterations are tissue-specific, arise from different mutagenic origin and differ in their co-mutation network to drive tumorigenesis.^7,10^

Multiple studies have demonstrated the potential influence of KRAS mutations on cancer patient prognoses, albeit with some conflicting findings. In general, KRAS mutations are linked to poorer prognosis, probably also influenced by tumor subtypes and co-occurring mutations.^10^ An important consideration regarding oncogenic KRAS mutations is the role of a co-existing wild-type (WT) allele. Studies using genetically engineered mouse models indicate that loss of the WT KRAS allele enhances tumorigenesis driven by the mutant one and influences the therapeutic outcome.^11–14^ This raises the question of whether the WT allele acts as a tumor suppressor and if it is the balance between WT and mutant allele that is crucial for tumorigenesis.

Similar to RAS, the MYC family ranks among the most extensively studied oncogene families. It encompasses c-MYC, N-MYC, and L-MYC, which govern critical cellular processes, including cell growth, proliferation, cell-cycle regulation, metabolism, and apoptosis.^15^ MYC was discovered over 40 years ago and is estimated to contribute to at least 70% of all human cancers, including prostate, breast, colon, ovarian and uterus, myeloid leukemia, lymphomas, small-cell lung carcinomas (SCLC), and neuroblastoma, among others, most of which are aggressive and respond poorly to the current therapies.^3,16^ However, unlike other common oncogenes, *MYC* is seldom mutated in cancer.^3^ Nevertheless, it is overexpressed in most human tumors, regulating the transcription of thousands of genes, either directly or indirectly.^17,18^ Notably, *MYC* holds the distinction of being the first oncogene to be found amplified in tumor cells. Indeed, it is one of the most frequently amplified genes in human cancer,^19^ especially in breast, ovarian and squamous cell lung cancer.^3^ In breast cancer, two of the largest performed genomic studies identified MYC amplifications in approximately 25% of patients. In this context, MYC amplification and activation of the MYC pathway are hallmark features of the basal subtype, with MYC amplification observed in 55.6% of cases and associated with aggressive disease and poor prognosis.^3^ In ovarian and endometrial cancers, TCGA analyses showed that *MYC* was amplified in 30.7% and 10.8% of tumors, respectively.^3^ In other genomic analyses in NSCLC,^20^ renal clear cell carcinoma^21^ and PDAC^22^ patients, MYC amplification was observed in 31%, 23% and 14%, respectively.

Besides being amplified, MYC is frequently involved in translocation events, such as within immunoglobulin loci in diffuse large B-cell lymphoma, Burkitt’s lymphoma and multiple myeloma, as well as T-cell receptor loci in T-cell acute lymphoblastic leukemia.^23^ In addition, multiple signaling pathways have been identified to modulate *MYC* gene expression, leading to *MYC* dysregulation in the absence of translocation or amplification.^3^

Strong evidence supports aberrant MYC expression as a driver of both tumor initiation and maintenance and is associated with all the hallmark features of cancer.^24–26^ Besides being a renowned master regulator of cell proliferation and survival pathways, MYC is also implicated in many resistance mechanisms to targeted therapies.^27^ Consequently, the MYC pathway, along with MYC itself, has been regarded as a promising target for cancer therapy for several years.

Importantly, MYC and RAS interplay is the paradigm of oncogene cooperation,^28^ starting from the first demonstration of their interdependence in 1983,^29^ until the most recent data on their joint role in immune suppression.^30^ Of note, MYC operates downstream of the KRAS pathway, and driver mutations in KRAS can trigger the amplification of MYC signaling,^31,32^ establishing a clear interdependence between these two oncogenes. Indeed, this partnership has long been regarded as a prime example of cooperating oncogenes,^29^ and it beckons the question of how precisely KRAS and MYC collaborate in the multifaceted processes that underlie oncogenesis.

In this review, we delve into their intricate interplay, providing a comprehensive overview of the molecular mechanisms underlying their collaborative effects on the pathogenesis of cancer, their impact on the tumor immune microenvironment, and their significant role in the development of therapeutic resistance. By deciphering the multifaceted dynamics of MYC and RAS cooperation, this review seeks to provide insights into potential therapeutic vulnerabilities and opportunities for developing novel precision medicine approaches to combat malignancies driven by MYC and KRAS dysregulation, able to improve treatment outcomes and address the challenge of therapy resistance in cancer.

## The oncogene cooperation concept

A clear genetic cause of cancer often lies on the mutation or aberrant expression of normal genes, which, consequently, transition from proto-oncogenes to oncogenes. Proto-oncogenes, in general, play essential roles in maintaining normal cellular homeostasis, regulating growth, proliferation and survival processes that, when deregulated in cancer cells, can confer a competitive edge over normal cells. In addition, mutations in these proto-oncogenes can, in many instances, lead to inhibition of cell death, replicative immortality or direct induction of angiogenesis.^25^ Since the first oncogene (*src*) was described in the 1970s,^33^ hundreds of cancer genes have been reported, accounting so far for more than 1% of all the genes in the human genome.^34,35^

However, it is important to note that most oncogenes lack the capacity to drive cell proliferation by themselves, having evolved with intrinsic and self-limiting safe mechanisms, which engage evolutionary dead-ends such as apoptosis or senescence programs when they are overexpressed. An example of this phenomenon is represented by both *RAS* and *MYC* themselves. Indeed, ectopic expression of activated RAS was shown to induce cell cycle withdrawal phenotypically identical to replicative senescence,^36^ which needs to be prevented for continuous and proficient cancer cell proliferation. In the case of *MYC*, cooperative mutations that suppress apoptosis (one of the many consequences of its deregulated expression) become essential to unleash its full transforming potential.^37^ Hence, both oncogenes require molecular mechanisms able to disable the intrinsic handbrakes in their tumorigenic paths. In this review, we will focus on the cooperative mechanisms that promote tumor initiation and maintenance. This phenomenon, characterized by the interplay of various oncogenes and their complementary actions is referred to as oncogenic cooperation, a central concept in the complex landscape of cancer development and progression.

## The RAS oncogene: activation and signaling pathways

In the 1960s, Harvey et al. and Kristen et al. initially identified viruses that induced sarcoma in rats, naming them Ha-Ras and Ki-Ras, respectively.^38,39^ A few years later, the homologous transforming human genes were discovered as well.^40^ In humans, three distinct *RAS* genes encode four different RAS isoforms: *NRAS* (*neuroblastoma RAS* viral oncogene homolog), *HRAS* (*Harvey rat sarcoma* viral oncogene homolog), and *KRAS*, whose RNA can be alternatively spliced into *KRAS4A* and *KRAS4B*.^41^ In mice, *KRAS4B* is the most expressed *RAS* isoform, representing 60 to 99% of all *RAS* transcripts, followed by *NRAS*, *KRAS4A* and *HRAS*.^42^ Surprisingly, though, the ranking of RAS protein isoforms levels is instead KRAS4B > HRAS > NRAS, highlighting differences between mRNA and protein expression.^43^ Due to variations in their prevalence, the roles of the two different KRAS isoforms have been extensively studied. In a wide panel of human cancer cell lines, KRAS4B was on average significantly more expressed than KRAS4A, although different ratios were observed across tissues.^43^ Consequently, KRAS4B has been the most studied isoform, but KRAS4A transcriptional activity is also widespread in cancer.^44^ Both isoforms exhibit distinct subcellular trafficking and isoform-dependent effector specificity, which may explain their non-overlapping functions in cancer.^45,46^

RAS proteins are small GTPases that play a critical role in cellular signaling pathways, which drive cell growth, proliferation and survival.^47^ The precise mechanisms by which RAS proteins function are still not fully understood, but their interaction with cellular membranes is essential for their action and intervention in RAS-driven diseases.^48^ The general notion is that they cycle between ON and OFF states during signal transduction, acting as binary switches. When bound to GTP, RAS proteins are active and facilitate signal transmission within cells. Conversely, when bound to GDP, they assume an inactive state, ceasing signaling activities.^4^ Since GDP is normally tightly bound and the intrinsic GTPase activity of RAS is poor, the transition from the stable and inactive state to the active state is triggered by guanine nucleotide exchange factors (GEFs), while the reversion to the inactive state is mediated by GTPase-activating proteins (GAPs).^49^ RAS proteins consist of a G domain and a C-terminal membrane targeting region, which play essential roles in their activity and interaction with cellular membranes. The G domain includes the switch I and II that bind guanosine nucleotides, enabling the GTPase activity of RAS, acting as the binary switch, cycling between ON and OFF states, and forms the binding interface for effector and regulatory RAS factors. On the other hand, a C-terminal membrane targeting region undergoes lipid modification, that enables the association of RAS proteins with cellular membranes.^50^

RAS signaling can be activated by a number of cellular receptors, being the most common ones the receptors tyrosine kinases (RTKs).^51^ Mitogen-activated signaling cascades initiate RAS activation through assembly of several adapter proteins like the growth factor receptor-bound protein 2 (GRB2), which, in turn, recruits the GEF Son of Seventless 1 (SOS1) that mediates the conversion of RAS from the inactive GDP-bound to the active GTP-bound state.^52^ In turn, GAPs such as the P120GASP/RASA1 and neurofibromin (NF1), among others, are well-known RAS GAPs responsible for transitioning RAS from its active to its inactive state.^53^ Importantly, the recruitment of adapter proteins and the RAS GTPase cycle can be regulated by phosphorylation. For instance, the v-Src kinase facilitates the recruitment of the GRB2/SOS complex, enabling RAS activation.^54^ Conversely, Src negatively regulates RAS by binding to and phosphorylating GTP-bound RAS in Tyrosine 32 (Tyr32). This impairs the interaction with effectors and increases GAPs binding.^55^ On this matter, Kano et al. shed light on the regulation of the KRAS GTPase cycle and identified Tyr32 and Tyr64 in the KRAS protein, within the switch I and II regions, respectively, as targeted residues of Src. These phosphorylations alter the conformation of the G domain, so that phosphorylated KRAS remains GTP-bound and its GTPase cycle is stalled, while its interaction with downstream effectors is impaired.^56^ The Src-induced phosphorylation of KRAS can in turn be reverted by the Src homology 2-containing protein tyrosine phosphatase 2 (SHP2) to rapidly activate KRAS and maintain the dynamic KRAS GTPase cycle.^56^ In addition, SHP2 dephosphorylates the adapter protein GAB1 and the epidermal growth factor receptor (EGFR), which sustains RAS activation.^57,58^ Accordingly, other studies have demonstrated that SHP2 is necessary in mutant KRAS-driven cancers.^59,60^ More recently, the protein-tyrosine phosphatase non-receptor type 2 (PTPN2) was also found to negatively regulate tyrosine phosphorylation of KRAS and its plasma membrane association.^61^

**Fig. 1:**
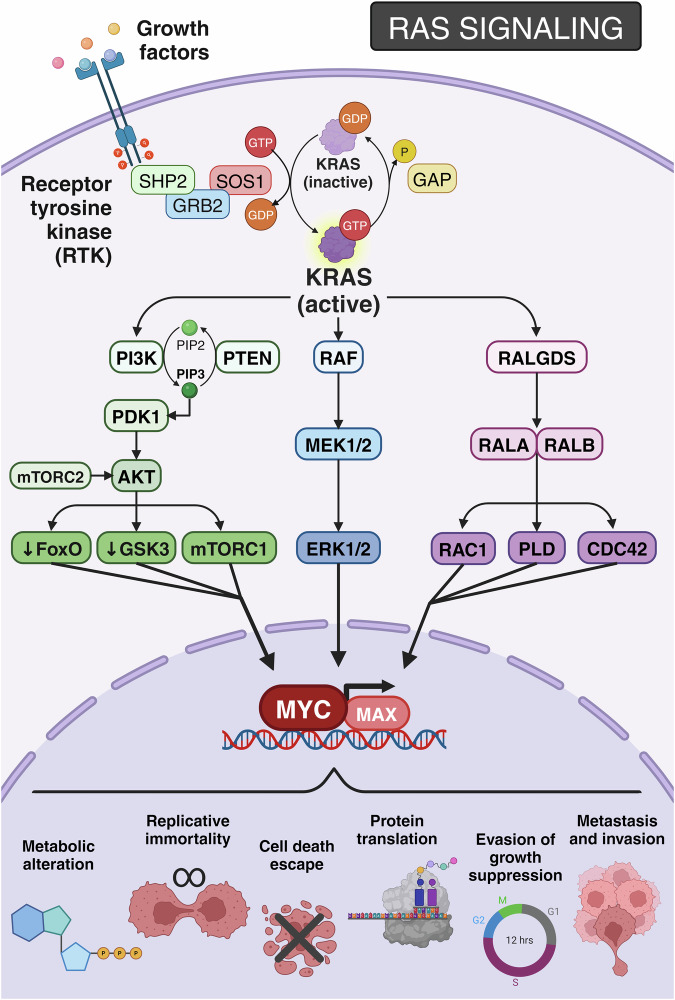
RAS signaling pathway. Various soluble growth factors activate receptor tyrosine kinases (RTK) on the surface of tumor cells. The recruitment of several proteins, such as Src homology region 2 domain-containing phosphatase-2 (SHP2) and growth factor receptor-bound protein 2 (GRB2), can initiate the recruitment of a guanine nucleotide exchange factor, like Son of Seventless 1 (SOS1), facilitating the conversion of KRAS from its inactive GDP-bound state to an active GTP-bound state. This dynamic is reversible through the engagement of GTPase-activating proteins (GAPs). The three most prevalent KRAS-activated pathways are: 1. PI3K/PDK1/AKT pathway, in which PI3K phosphorylates PIP2 to PIP3, attracting PDK1 that, together with mTORC2, activates AKT, regulating FoxO family, GSK3 and mTORC1; 2. RAF/MEK1/2/ERK1/2 pathway; and 3. RALGDS/RALA/RALB pathway, which activates the effectors RAC1, CDC42 and PLD. All these pathways culminate in the activation of MYC target genes, propelling key cancer hallmarks such as metabolic reprograming, replicative immortality, cell death escape, protein translation, resistance to growth suppression, metastasis and invasion. Created with BioRender.com

Despite RAS proteins activation being tightly controlled, and mutations in the *RAS* genes or their regulators can render RAS proteins persistently active. Once turned on, RAS activates multiple downstream pathways which drive cell proliferation, differentiation and promote survival^62–65^ (Fig. 1). To date, more than 10 RAS downstream signaling pathways have been described,^66^ and recent studies have identified up to 50 proteins containing RAS-interacting domains in the human proteome.^67^

The RAF-MEK-ERK axis represents a primary mitogen-activated protein kinase (MAPK) pathway and is one of the most extensively described downstream RAS pathways. Upon RAS activation, the rapidly accelerated fibrosarcoma (RAF) kinase is recruited to the plasma membrane via two RAS-binding sites. Subsequently, RAF undergoes activation through phosphorylation by membrane-associated kinases.^68,69^ Activated RAF then phosphorylates the mitogen-activated protein kinase 1/2 (MEK1/2), which further phosphorylates and activates the extracellular signal-regulated kinase 1/2 (ERK 1/2).^70^ Ultimately, ERK1/2 translocates to the nucleus to activate various nuclear target proteins, including c-MYC, which promotes cell growth, differentiation, survival, and invasion.^65,71,72^

Another well-established RAS effector is the phosphatidylinositol-3-OH kinase (PI3K), which directly interacts with RAS-GTP.^73^ The canonical pathway starts with activated PI3K, which phosphorylates the phosphatidylinositol (4,5)-biphosphate (PIP2) into phosphatidylinositol (3,4,5)-triphosphate (PIP3), allowing interaction with the phosphatidylinositol-dependent kinase-1 (PDK1).^74^ This process can be negatively regulated by phosphatases, such as the tumor suppressor 3-phosphatase and tensin homolog (PTEN).^75^ In fact, loss of PTEN and KRAS overexpression synergize during tumor development in mouse models of CRC and PDAC.^76,77^ PIP3 then recruits inactive PKB/AKT to the plasma membrane, where it is generally phosphorylated and activated by both PDK1^78^ and the mammalian target of rapamycin complex 2 (mTORC2).^79^ The main downstream effects of AKT include the inhibition of both the glycogen synthase kinase 3 (GSK3) and the Forkhead Box O (FoxO) family, and the activation of the mTORC1 complex.^80^ Altogether, AKT promotes pathways related to survival, anabolic metabolism and proliferation.^63^

The RAL (RAS-Like) signaling pathway has also been implicated in regulating cell proliferation and survival as major effector of RAS^62^ interacting with several RAL-GEFs, such as the RAL guanine nucleotide dissociation stimulator (RALGDS), and activating the RAL GTPases RALA and RALB.^81^ Although RALA is pivotal for tumor growth and RALB holds importance in tumor metastasis and invasion, both isoforms can act redundantly in cancer development. However, in most cancer models, RALA appears to be the predominant RAL isoform responsible for tumor progression and metastasis.^82^ Interestingly, some studies demonstrated a cooperative interaction between RAS and PI3K pathway to activate the RALGDS through PDK1.^83,84^ Once activated, RAL interacts with a spectrum of downstream effectors including RAC-1, CDC42 phospholipase D (PLD).^85^

The initial explorations into oncogenic mechanisms driven by RAS mutations linked point mutations in the RAS protein with a decrease in its intrinsic GTPase activity.^86^ Later, it was described that GAP-stimulated GTPase activity of RAS was impaired in the presence of G12V and G12A mutations.^87^ Collectively, these mechanisms were shown to promote the GTP-bound activated state of RAS. When focusing on the KRAS protein, most of KRAS mutants exhibit a reduced rate of GAP-stimulated hydrolysis compared to WT. Among the variety of KRAS mutations, disparity regarding intrinsic and GAP-stimulated hydrolytic capacity was also observed.^88^

Deciphering which of the RAS downstream pathways is primarily activated in a particular tumor could enhance the efficacy of targeted therapies, since different RAS isoforms differ in their ability to activate specific signaling pathways to induce transformation.^89,90^ In this regard, Muñoz-Maldonado and colleagues conducted a comprehensive review of the differences among RAS isoforms and point mutations in signaling, transcriptomic, proteomic and metabolomic profiles. Accordingly, each isoform and mutation displayed differences in terms of transforming ability, GTP binding, anchorage-independent growth and metastatic capacity.^91^ Specifically, the interaction of different KRAS mutants with RAF1 revealed affinity variabilities. While mutations at positions 13 and 61, as well as G12 to A or C displayed a slight decrease in affinity when compared to KRAS WT, substitutions of D, V or R in position 12 had a more significant fold decrease.^88^ In mouse tumors, KRAS G12V, but not G12D was found to phosphorylate ERK through RAF1.^92^ In the same study, the authors demonstrated that while both KRAS G12V and G12D could interact directly with PI3K, G12V mutation failed to trigger AKT activation, whereas G12D notably induced it. Conversely, in the colon, the latter showed a decrease in AKT phosphorylation compared to WT.^93^ In NSCLC cancer cell lines, G12D mutations also activated PI3K an MAPK signaling, while G12C and G12V showed an increase in RAL signaling along with a decrease in AKT activation.^94^ Likewise, when compared to WT KRAS, mutant KRAS had a higher expression of RALA and RALB, with G12C being more dependent on the RAL pathway to induce cell growth.^95^ However, in mouse models, genetic deletion of both RAL isoforms impaired tumor formation in a model of Kras^G12D^- driven NSCLC,^96^ and deletion of ERK and MEK kinases reduced tumorigenesis in a Kras^G12V^- driven NSCLC.^97^ Notably, tumors from a mouse model of Kras^G12C^ lung adenocarcinoma showed higher ERK1/2 phosphorylation and responded better to MEK inhibition compared to Kras^G12D^ lung tumors.^98^ Overall, these studies, along with others reviewed elsewhere, suggest that the specific downstream signaling pathway triggered by KRAS activation depends on the tumor type and its specific context.^91^

Importantly, the mutation profile of the KRAS protein has also been linked to clinical outcomes, a phenomenon primarily studied in NSCLC, PDAC and CRC, where KRAS mutations are prevalent.^6^ For example, in advanced NSCLC, mutant KRAS was not associated with lower progression-free survival (PFS) and overall survival (OS) in patients treated with standard first-line platinum-based chemotherapy.^99^ However, KRAS mutant patients in the same stage showed improved OS with immune checkpoint inhibitors (ICIs) compared to KRAS WT patients.^100^ Regarding the relevance of the distinct KRAS point mutations for clinical outcome in NSCLC, in a refractory cohort, patients harboring mutant KRAS-G12C or G12V displayed worse PFS compared to those with other mutant KRAS proteins or WT.^94^ Moreover, G12C mutations, but not other substitutions, showed a significant reduced PFS in response to chemotherapy.^101^ However, in a study comparing various chemotherapy regimens, NSCLC patients with G12V mutations demonstrated a higher overall response rate (ORR) when treated with taxanes compared to G12C or G12D mutations.^102^ Another study by Renaud et al. in advanced NSCLC similarly identified the G12V mutation as the most responsive to taxanes, whereas it exhibited the worst response rate to pemetrexed.^103^

In metastatic CRC cancer (mCRC), patients with KRAS mutations showed lower PFS and OS compared to those with WT KRAS, displaying poorer prognosis.^104^ In chemotherapy-refractory mCRC, patients carrying KRAS-G13D treated with the EGFR inhibitor cetuximab plus chemotherapy had longer PFS and OS compared to other mutant KRAS variants.^105^ In patients treated with the anti-angiogenic antibody bevacizumab, it was observed that KRAS mutant patients exhibited inferior PFS and OS compared to those with WT KRAS, and this effect was specifically attributed to G12V and G12A mutations.^106^

In PDAC, the impact of mutant KRAS is somewhat contradictory. In one study assessing the OS of patients with metastatic or locally advanced disease, no differences were reported between mutant and WT KRAS patients. However, patients harboring a G12D mutation had a significant shorter OS compared to WT and other KRAS mutations.^107^ In contrast, Yousef et al. recently demonstrated that KRAS mutant patients, especially those harboring mutations in Q61 and G12D, had an inferior OS compared to WT. Similar results were obtained in metastatic patients, with G12D mutation displaying a significantly worse OS relative to KRAS WT. Accordingly, this mutation was enriched in patients with metastatic disease compared to those with localized disease.^108^ Similarly, patients with resectable PDAC harboring a G12D mutation had reduced OS compared to other KRAS mutations and WT.^109^

Collectively, these studies, along with others, demonstrate that different mutations in KRAS exhibit disparities in terms of signaling, behavior, and clinical outcomes. Moreover, the impact of each KRAS mutation appears notably contingent on the tumor type. Consequently, comprehending the interplay between KRAS and its downstream effectors proves pivotal to the development of more effective targeted therapies, as well as the assessment of the mutational profile when determining treatment strategies. Of note, the extent of cooperation between various KRAS mutants and MYC remains an enigma yet to be elucidated.

## The MYC oncogene: family, function, and oncogenic activation

The *MYC* oncogene family consists of *MYC* (c-MYC), *MYCN* (N-MYC), and *MYCL* (L-MYC). These three paralogs share a similar function but exhibit distinct expression patterns and tissue-specificities, with c-MYC being the most widely expressed across different tissue types. Each *MYC* paralog is situated on a different chromosome and expressed at distinct timings and locations during development.^110,111^ c-MYC is the most expressed both during tissue development and in various tumors (hematological and solid). N-MYC is expressed in neural tissue and early hematopoietic development, and is deregulated in different cancer types, in particular neuroblastoma, where it can functionally substitute for c-MYC. In neuroblastoma, *MYCN* amplification is a marker of aggressive tumors, characterized by unfavorable prognosis and diminished survival rates, and it is used for risk stratification classifications.^112^ Finally, L-MYC has the most restricted pattern of tissue expression, being mainly present in the lungs and particularly overexpressed in SCLC, although it is considered to have lower transforming activity compared to the other family members.^113,114^ It is worth mentioning that recent genome-sequencing studies have unveiled a broader involvement of both N-MYC and L-MYC in various cancers beyond their conventional associations. As a general rule, the majority of human cancers exhibit genetic activation of at least one of the MYC family members.^16^

All MYC proteins (MYC, from now on) control different cellular programs that regulate both cell-autonomous biology as well as the host’s immune system and tumor microenvironment (TME), primarily acting as an essential transcription factor that impacts diverse cellular processes, such as cell growth, cell cycle, differentiation, apoptosis, angiogenesis, metabolism, DNA repair, protein translation, mitochondrial biogenesis, immune response and stemness.^16,115,116^

All MYC family members share three domain-type structures:

(i) An N-terminal region encompassing highly conserved transcriptional regulation elements known as “MYC boxes” (MB). Within this domain, there are two important phosphorylation sites, Threonine 58 (T58) and Serine 62 (S62). Their sequential and ordered phosphorylation is essential for MYC correct and timely function. With a very simple distinction, S62 phosphorylation enhances MYC stability, while T58 phosphorylation targets MYC for degradation.^117^
(ii) A central region, which also contains MBs, implicated in nuclear localization as well as protein stability.
(iii) A C-terminus region comprising the basic Helix–Loop–Helix Leucine Zipper (bHLHZ) domain, involved in binding to DNA and protein dimerization.^18^

Importantly, MYC is generally unstructured until it heterodimerizes with its obligate partner MAX (MYC-associated protein X).^118,119^ This interaction with MAX enables MYC to recognize the canonical Enhancer box (E-Box) CACG/ATG elements in the enhancers and promoters of target genes. This recognition leads to the recruitment of multiple transcriptional cofactors via its Transactivation Domain (TAD), stimulating RNA polymerases I, II, and III, switching on transcription of at least 15% of all genes, encoding both proteins and non-coding RNA products.^120–122^ This pleiotropic effect renders MYC a master transcriptional regulator. Furthermore, the binding of MYC/MAX heterodimers to E-boxes induces dynamic changes in chromatin structure through upregulation of histone acetyltransferases, which opens chromatin and allows the access of the DNA Polymerase II.^123,124^ This comprehensive transcriptional regulation initiates multiple processes including cell cycle progression, DNA replication, ribosome and mitochondrial biogenesis, and metabolism.

MYC and MAX belong to a bigger network of transcription factors called the Proximal MYC Network (PMN), which comprises bHLHZ proteins that dimerize with each other and recognize DNA on similar consensus sequences.^115^ Other members of the PMN include MXD1 (MAD1), MXI1 (MXD2, MAD2), MXD3 (MAD3), MXD4 (MAD4), MGA, MNT, MLX, MLXIP (MONDOA), and MLXIPL (CHREBP).^18^ MAX is the central node of the PMN and, in addition to dimerizing with MYC paralogs, is able to dimerize with itself and with other bHLHZ proteins. For instance, MAX can form heterodimers with proteins of the MXD family (MXD1, MXD3, MXD4), MAX-binding protein MNT and MAX gene-associated protein MGA. These complexes constitute functional antagonists of MYC and MYC-induced transcriptional activation.^115,125,126^

Although MYC is generally considered a transcriptional activator through the recruitment of coactivator partners, it can also repress the transcription of some target genes through the interaction with MYC-interacting zinc finger protein-1 (MIZ-1)^127–130^ and specificity protein 1 (SP1).^126,131^ Of note, besides promoting cell proliferation, MYC also plays a crucial role in sensitizing cells to apoptosis, preventing MYC-dependent uncontrolled cell growth. MYC-induced apoptosis occurs through various mechanisms, including indirect activation of the tumor suppressor ARF,^132^ p53 stabilization^133^ or through MIZ-1-mediated transrepression, where the interaction between MYC and MIZ-1 results in the repression of the anti-apoptotic BCL-2 gene,^134^ required for MYC to induce cell death in specific cellular contexts.^135^

In physiological conditions, MYC expression is tightly regulated. In contrast to MAX that is constitutively expressed, MYC proteins are absent in quiescent cells. MYC expression is instead rapidly induced in response to specific biological conditions, like those triggered by mitogenic signals.^136,137^ Both *MYC* mRNA and protein have a short half-life in the order of 15–20 min, ensuring precise and dynamic control of MYC activities.^15^ This switch is tightly regulated at the transcriptional and post-transcriptional levels by multiple extracellular and intracellular signals.^138^ Additionally, at the protein level, MYC undergoes activation, stabilization, or degradation through multiple mechanisms, including phosphorylation, ubiquitination, sumoylation and acetylation.^139^ Another layer of MYC regulation indirectly takes place through the modulation of other proteins within the PMN.^114^ Consequently, under non-pathological conditions, MYC exhibits ubiquitous expression during embryonic development, while in adults, it is primarily present in proliferative and regenerating tissues, such as during wound healing.^140^

**Fig. 2:**
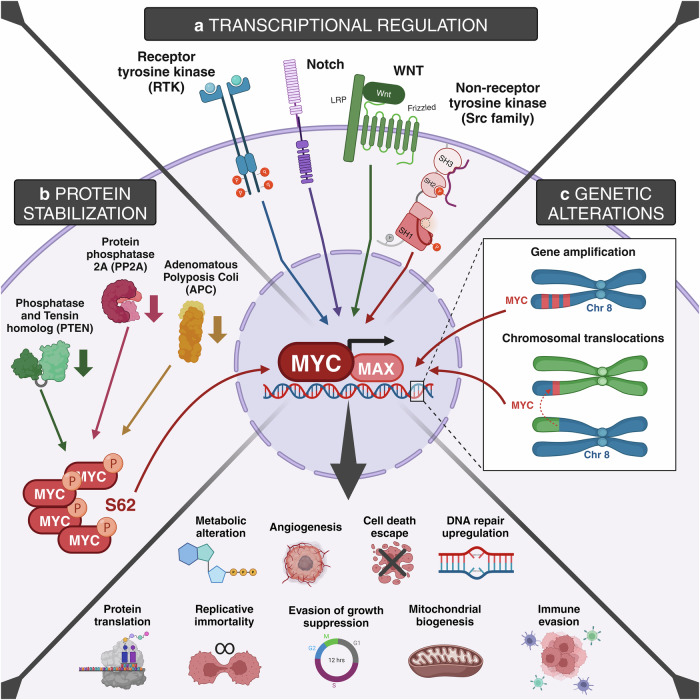
MYC activating pathways. Oncogenic MYC activation can occur through three different mechanisms. **a** Transcriptional regulation: the activation of upstream receptors, including receptor tyrosine kinase (RTK), NOTCH, WNT, and non-receptor tyrosine kinase (Src family) can ultimately result in aberrant MYC activation. **b** Protein stabilization: inactivation of tumor suppressor genes such as Adenomatous Polyposis Coli (APC), Phosphatase and Tensin homolog (PTEN), and Protein Phosphatase 2A (PP2A) can lead to MYC activation, stabilization, and accumulation. **c** Genetic alterations: intrinsic gene amplification or chromosomal translocations within chromosome 8 can cause abnormal MYC activation. Given the pivotal role of MYC in several crucial pathways, these processes culminate in various cancer hallmarks, including metabolic alterations, angiogenesis, cell death escape, upregulation of DNA repair mechanisms, protein translation, replicative immortality, evasion of growth suppression, mitochondrial biogenesis, and immune evasion. Created with BioRender.com

As mentioned in the introduction, MYC deregulation has been reported in approximately 70% of all human cancers. In all instances, MYC deregulation correlates with highly aggressive tumors, which show poor response to treatment, linking MYC overexpression with poor prognosis.^111,141^ MYC overexpression is due to gene amplification in 15–20% of the patients, while, in the rest, is usually related to induction/stabilization by upstream oncogenic signals. In a nutshell, MYC activation in cancer can occur through multiple mechanisms, including genetic, epistatic, epigenetic, post-translational, and direct genetic activation, which can differ among cancer types. Genetic activation may occur through processes like the insertion of upstream enhancers by retroviruses or through the activation of various upstream oncogenic signaling transduction pathways, such as WNT-β-catenin, SRC, RTKs, and NOTCH.^16,142^ Inactivation of tumor suppressor genes such as Adenomatous Polyposis Coli (APC), PTEN, and Protein Phosphatase 2A (PP2A), can also lead to MYC activation, stabilization and accumulation.^143,144^ All these alterations result in higher or aberrantly sustained levels of MYC protein^145^ (Fig. 2). Importantly, even minor deregulated tonic signaling through MYC (e.g. 2-fold relative to normal levels) is enough to support tumorigenic processes.^146^ In such circumstances, oncogenic MYC interacts with promoters containing canonical E-boxes, amplifying the expression of established gene programs. Simultaneously, it also engages enhancers with lower-affinity, non-canonical E-boxes in a dose-dependent fashion, thereby initiating the aberrant regulation of previously silent genes.^147,148^ Hence, even in scenarios where MYC is not the driving oncogenic lesion, it serves as a nexus for integrating both extracellular and intracellular oncogenic signals.

Ample evidence demonstrates that MYC is involved in the initiation, maintenance and progression of several cancer types and is associated with all the hallmark features of cancer, including proliferation, self-renewal, cell survival, genomic instability, metabolism, invasiveness as well as angiogenesis and immune evasion.^25,26^ However, as previously mentioned, its activation alone generally is not sufficient to induce tumor progression, which requires other cooperative oncogenic lesions.^26^

## Interplay between RAS and MYC oncogenes in a cancer cell

**Fig. 3:**
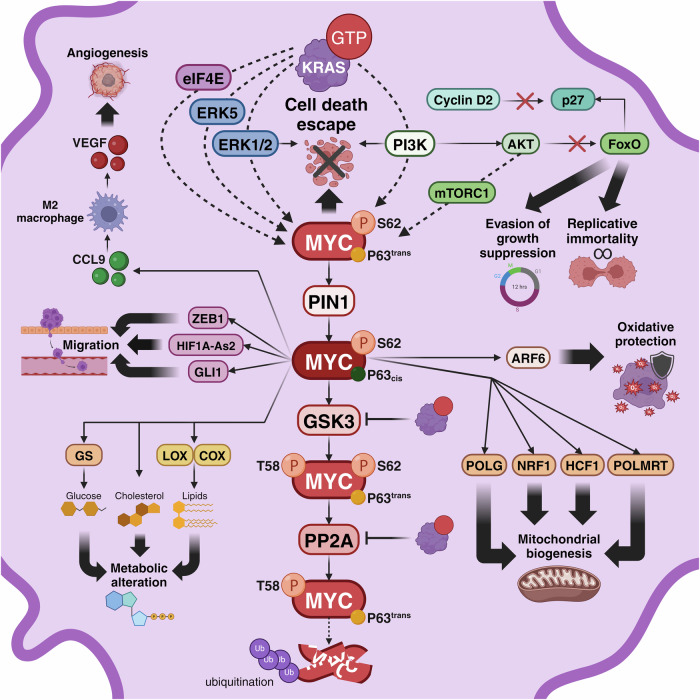
Molecular mechanisms of oncogenic KRAS and MYC cooperation. Active KRAS initiates the phosphorylation of Serine 62 (S62) in the MYC protein via the extracellular signal-regulated kinase 1/2 (ERK1/2) and phosphoinositide 3-kinase (PI3K) pathways. KRAS can also regulate MYC through ERK5 and eukaryotic translation initiation factor 4E (eIF4E). Subsequently, the peptidylprolyl-isomerase 1 (PIN1) enzyme facilitates the cis-trans isomerization of Proline 63 (P63). Following this, glycogen synthase kinase-3 (GSK3) phosphorylates Threonine 58 (T58), followed by another round of cis-trans isomerization of P63. Dephosphorylation of S62 is orchestrated by protein phosphatase 2A (PP2A), ultimately marking MYC for ubiquitination. Both GSK3 and PP2A function are inhibited by active KRAS, promoting MYC stabilization. Beyond this principal pathway, various molecules contribute to MYC/RAS cooperation, triggering numerous hallmarks of cancer. Cyclin D2 experiences upregulation in the presence of oncogenic KRAS and MYC, inhibiting p27. PI3K indirectly inhibits p27 by upregulating protein kinase B (AKT) and inhibiting Forkhead box O (FoxO) proteins, thereby facilitating the evasion of growth suppression and replicative immortality. Moreover, the combined action of PI3K with ERK and MYC induces cell death escape. In addition, tumors expressing oncogenic KRAS and MYC release chemokine (C-C motif) ligand 9 (CCL9), activating M2 macrophages to secrete vascular endothelial growth factor (VEGF) and ultimately promoting angiogenesis. In parallel, biomolecules such as glucose, cholesterol, and lipids depend on MYC-induced enzymes like glutamine synthetase (GS), lipoxygenase (LOX), and cyclooxygenase (COX), resulting in metabolic alterations. Moreover, MYC regulates several genes and enzymes responsible of mitochondrial biogenesis and metabolism, such as RNA polymerase mitochondrial (POLMRT), DNA polymerases (POLG), nuclear respiratory factor (NRF-1) and host cell factor 1 (HCF1). In addition, KRAS and MYC interaction activates ADP-ribosylation factor 6 (ARF6), conferring oxidative protection. Finally, oncogenic KRAS and MYC elevate the levels of long non-coding RNA HIF1A-As2, transcription factors zinc finger E-box binding homeobox 1 (ZEB1) and zinc finger protein GLI1, contributing to invasion and migration. Created with BioRender.com

Since the discovery of MYC and KRAS cooperation, their joint role in multiple aspects of tumorigenesis has been a topic of debate. With their far-reaching impact on every hallmark of cancer, RAS and MYC synergistically amplify the core mechanisms underlying tumorigenesis (Fig. 3). Here, we summarize the intricate molecular pathways fostered by the interplay between oncogenic RAS and MYC.

### MYC protein stabilization by RAS

A pivotal paradigm underlying MYC and RAS cooperation is the well-established MYC protein stabilization by RAS and its direct effector pathways.

To start with, KRAS signaling promotes c-MYC mRNA translation through phosphorylation and activation of the eukaryotic translation initiation factor 4E (eIF4E) and mTORC1. In fact, inhibition of both pathways triggers the suppression of c-MYC protein, effectively reducing proliferation in a mouse model of KRAS-driven CRC.^149^ Likewise, inhibition of eIF4E also mitigates the overexpression of MYC in PDAC mutated in KRAS.^150^

In addition, following RAS/RAF/MEK signaling, ERK stabilizes the MYC protein through phosphorylation at S62.^142^ This step allows recognition by the peptidylprolyl cis/trans isomerase 1 (PIN1), which isomerizes Proline 63 (P63) to promote MYC recruitment to DNA. Notably, PIN1 levels can also be increased by RAS downstream effectors.^151^ Then, subsequent phosphorylation of MYC at T58 (pS62/pT58-MYC) by GSK3 allows a second isomerization at P63 by PIN1. This step enables the dephosphorylation of S62 by the protein phosphatase PP2A, which leads MYC to a degradation complex facilitated by the scaffold protein Axin1. This process can be impaired through inhibition of GSK3 by activation of the PI3K/AKT axis.^152^ Finally, the E3 ubiquitin-ligase FBXW7 drives MYC to proteasomal degradation.^153,154^ The KRAS-ERK axis can activate the inhibitor of apoptosis protein Survivin to inhibit both MYC autophagic degradation and its dephosphorylation through a CIP2A-dependent inhibition of PP2A.^155^ Accordingly, in PDAC cell lines, KRAS suppression causes MYC protein degradation. Notably, while KRAS regulates MYC protein stability through an ERK1/2-dependent mechanism, inhibition of this kinase activates a compensatory pathway through the non-canonical ERK5 to stabilize MYC.^156^ Long-term inhibition of ERK in KRAS-driven pancreatic cancer was also related to MYC proteasomal degradation, which induced a senescence-like phenotype. Importantly, sensitivity to ERK1/2 inhibition was correlated to MYC loss.^157^

Collectively, mutant KRAS, through downstream effectors, is involved in both MYC protein accumulation and stabilization by promoting MYC mRNA translation and preventing its proteosome degradation.

### Cell-intrinsic tumorigenic mechanisms induced by MYC and RAS cooperation

Multiple studies have explored the collaborative interplay between RAS and MYC in terms of cell-intrinsic tumorigenic mechanisms. As mentioned before, for example, while driving proliferation, RAS also induces senescence, a phenomenon often found in pre-neoplastic lesions.^36^ In this context, a potential positive selection condition could be caused by MYC upregulation of cell cycle genes and repression of p16 and p21, which altogether prevent RAS- and BRAF^V600E^-induced senescence.^158,159^ Similarly, MYC’s role as a potent oncogene is often hampered by cell sensitization to apoptosis.^160^ In this context, RAS-dependent activation of PI3K, through PKB/AKT^161^ and MEK/ERK^162^ can suppress MYC-induced apoptosis sustaining tumor growth. Indeed, pharmacological inhibition of GSK3, which is also negatively regulated by RAS/PI3K/AKT, partly depends on MYC to induce apoptosis in KRAS-driven human cell lines.^163^ Conversely, in KRAS mutant NSCLC and CRC cell lines, chemotherapy-induced cytotoxicity appears to be dependent on MYC inhibition.^31^ These studies imply context-dependent feedback between both oncogenes in regulating apoptosis induction.

Another mechanism of cooperation between MYC and RAS is their sustained proliferative signaling, which relies on efficient cell cycle machinery. One of the cell cycle control tools is the cyclin-dependent kinase inhibitor p27. Enhanced activation of p27^164^ and downregulation of cyclin D2^165^ by FoxO transcription factors inhibit cell cycle progression. However, when MYC is activated, it upregulates cyclin D2, which, in turn, sequesters p27 and allows cell cycle progression and cell proliferation.^166^ In this same context, parallel activation of KRAS activates PI3K, enabling the AKT-dependent inactivation of FoxO, driving the expression of MYC and its target genes, including cyclin D2, triggering cell proliferation and transformation.^167^ The collaboration between MYC and RAS has also been associated with accumulation of cyclin E/CDK2 and the E2F transcription factor,^168^ and with the cooperative regulation of cyclin D1-CDK4 complex to induce proliferation through compensatory mechanisms between these oncogenes.^169^

MYC and RAS interaction may also have an impact on cancer cell migration. In fact, anchorage deprivation triggers p27 upregulation and cell cycle arrest, which can normally be overcome by RAS activation. However, even a slight reduction of MYC expression has been shown to hinder RAS capacity to avoid p27 activation.^170^ During cell migration, in addition, RAS and RHO signaling collectively drive cell motility^171^ and MYC cooperates with RhoA/Rock, aiding stress fiber and focal adhesion disassembly, promoting invasion of transformed cells.^172^ In NSCLC, specifically, it has been shown that RAS-induced MYC activation generates a double-positive feedback loop with the lncRNA HIF1A-As2, promoting proliferation, EMT, and metastasis.^173^ In another study in lung modified KRAS-G12V cells, MYC induced the transcriptional factor ZEB1 to promote a metastatic phenotype.^174^ This is in line with what was seen in human colon carcinomas, where MYC inhibition was shown to downregulate GLI1, a transcription factor driving metastatic transition. Notably, oncogenic KRAS enhances GLI1 activity too.^175^ Both oncogenes have also been implicated in inducing features of EMT in breast cancer, cooperatively promoting gene expression profiles associated with invasion.^176^

MYC was also shown to facilitate the conversion of precancerous to cancerous cells in KRAS-G12D mutant fibroblasts and control the generation of self-renewing metastatic pancreatic cancer stem cells.^177^ In fact, in the context of KRAS-mutant PDAC, MYC promotes ductal-neuroendrocrine lineage plasticity, correlating with poorer survival and chemotherapy resistance.^178^ Additionally, it was recently described that in KRAS-driven primary liver cancer (PLC), MYC impacts lineage commitment to hepatocellular carcinoma (HCC) or intrahepatic cholangiocarcinoma (iCCA). Interestingly, this phenomenon depends on MYC levels, which modulate the epigenetic landscape and regulate the transcriptional activity of FOXA1, FOXA2 and ETS1.^179^ Apart from their cooperation in commonly mutated cancers, Pajovic and collegues also identified epigenetic activation of the RAS/MYC axis in tumors mutated in Histone H3 lysine 27 (H3K27M).^180^

### MYC and RAS metabolic reprogramming

Cancer metabolism plays a pivotal role in cellular transformation, given the frequent occurrence of metabolic stress in tumors.^181^ Metabolic rewiring, a crucial hallmark of cancer cells, is tightly regulated by KRAS in various tumor types such as NSCLC, CRC and PDAC,^182–185^ and this metabolic reprogramming is often accompanied by the concomitant reprogramming of the TME.^186^ In KRAS mutant PDAC, for instance, paracrine signaling appears to play a pivotal role in KRAS-driven metabolic changes.^187^ In addition to rewiring glucose and glutamine metabolism, KRAS also regulates lipid metabolism to enable cancer cells to adapt to metabolic stress.^188^ In this context, MYC also contributes to important metabolic changes, fostering metabolic pathways that supply bioenergetic substrates essential for tumor proliferation.^189^ This is particularly evident in the reprogramming of glutamine metabolism, a process vital in most cancer types.^190,191^

Numerous studies have demonstrated the role of MYC as a crucial stress adaptation factor in KRAS-driven cancers. In KRAS-mutant CRC, for example, MYC plays a fundamental role in regulating metabolic and transcriptomic adaptation to stress. Hence, CRC cells become reliant on elevated MYC, and its loss results in metabolic crisis and cell death.^192^ In PDAC, the KRAS/MAPK pathway, significantly alters glucose metabolism. Interestingly, knocking down MYC in this context leads to a substantial decrease in the expression of metabolic genes crucial for glycolysis, the hexosamine biosynthetic pathway (HBP), and the nonoxidative pentose phosphate pathway (PPP).^193^ Importantly, KRAS-driven tumors have also been described as glutamine-dependent.^194^ In the case of lung tumor cells from KRAS-G12D mice, increased MYC expression was shown to increase glutamine synthetase (GS) levels.^195^ Moreover, human KRAS-driven NSCLC cell lines demonstrated higher glutamine uptake and dependency compared to wild-type cells,^196^ and MYC role as a regulator of glutamine synthesis, uptake, and glutaminolysis through glutaminases has extensively been documented.^197^ Notably, stabilization of MYC protein through RAS downstream effectors can also prime cells for arginine deprivation in melanoma. In a study assessing the impact of an inhibitor of the argininosuccinate synthetase (AS), the enzyme responsible of arginine biosynthesis, RAS-induced ERK and AKT prompted adaptative MYC stabilization.^198^

However, the metabolic phenotype of KRAS-mutated cancer cells is also influenced by the TME. This is evident from variations in glucose and glutamine requirements observed between in vitro and in vivo context.^199^ Accordingly, in PDAC, KRAS promotes the expression of cytokine receptors that interact with cytokines secreted from the TME, which through JAK1-STAT6, directly upregulate MYC-dependent glycolysis.^187^

Lipid metabolism has also been described as a process crucial for tumor cell survival and proliferation.^200^ Hall et al. delved into the role of MYC and KRAS cooperation in a transgenic mouse model of KRAS-driven lung adenocarcinoma and showed that high MYC was linked to arachidonic acid-derived eicosanoid synthesis via lipoxygenase (LOX) and cyclooxygenase (COX) pathways, and inhibition of these enzymes reduced tumor progression.^201^ Moreover, MYC activity in the same mouse model enhanced lipid metabolism by increasing cholesterol intake and storage within lung tumors.^202^ Consistent with this, a recent study identified the phospholipid transporter PITPNC1 as being regulated by KRAS. Importantly, this leads to a MYC-dependent activation of mTOR, preventing autophagy and promoting tumorigenesis and metastasis.^203^ On the same line, MYC activity was also associated to metabolic adaptation after inhibition of eIF4E and mTOR pathways in KRAS-driven CRC. In this context, upregulated c-MYC promotes metabolic pathways such as oxidative phosphorylation and arachidonic acid metabolism.^149^ Cholesterol accumulation is also commonly observed in CRC. Interestingly, cholesterol was found to reduce MYC ubiquitination and degradation in KRAS-mutant cell lines of CRC, although the direct role of KRAS was not investigated.^204^

Mitochondrial metabolism and reactive oxygen species (ROS) generation are likewise critical for KRAS-driven tumorigenicity^205^ and, importantly, MYC is a well-known ROS generating oncogene.^206^ Moreover, the interaction among KRAS, MYC and the ADP-ribosylation factor 6 (ARF6) is well documented. Within this axis, mutant KRAS activates both MYC and ARF6. MYC is involved in mitochondrial biogenesis and oxidative phosphorylation (OXPHOS), while ARF6 confers protection from oxidative injury.^207^ Expression of the RNA polymerase mitochondrial (POLMRT), which is responsible of the mitochondrial genome transcription, is required for NSCLC progression.^208^ Crucial genes in mitochondrial replication and biogenesis, such as DNA polymerases and the nuclear respiratory factor 1 (NRF-1), are direct targets of MYC.^209^ Finally, the interaction between MYC and the host cell factor 1 (HCF1) has also been recently linked to mitochondrial function and ribosome biogenesis.^210^

### MYC and RAS induced angiogenesis

Intimately connected to the metabolic reprogramming of cancer cells to efficiently proliferate and adapt to reduced oxygen and nutrients availability is the capacity to enhance angiogenesis. In this context, both MYC and RAS play roles in promoting increased vascularity within tumors to ensure adequate oxygen and nutrient supply. The redundancy observed in the molecular mechanisms governing this process, particularly the upregulation of the vascular endothelial growth factor (VEGF), suggests a collaborative effort between these oncogenes in activating angiogenic pathways.^211,212^ This cooperative angiogenic interplay is also observed within the TME, where, for instance, in KRAS-G12D-driven lung adenocarcinoma, MYC-driven CCL9 expression recruits macrophages that release VEGF, thereby stimulating angiogenesis.^213^ In a similar model, MYC inhibition disrupted the array of chemokines, cytokines and, importantly, angiogenic factors, that participate in maintaining the lung cancer microenvironment.^214^

Overall, a range of studies highlights the direct role of MYC in regulating multiple cell- autonomous tumorigenic mechanisms in KRAS-driven cancers, indicating potential vulnerabilities under its cooperation.

## Effect of the interplay between RAS and MYC in the tumor immune microenvironment

**Fig. 4:**
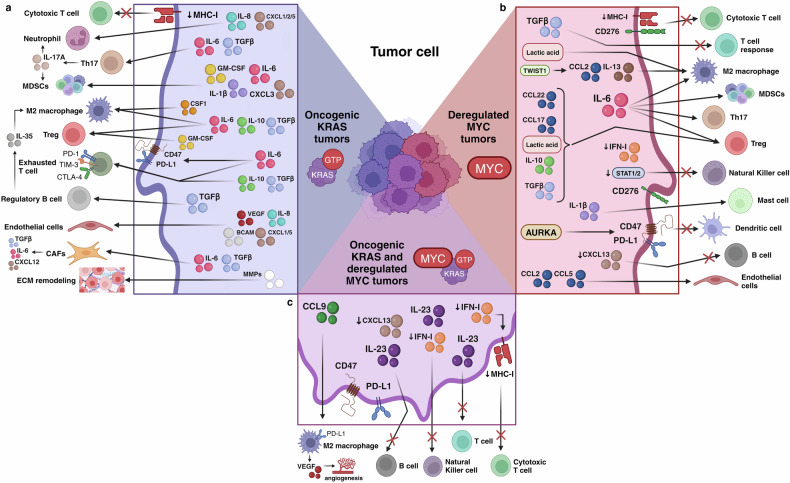
Influence of KRAS mutation and/or MYC deregulation on the tumor immune microenvironment. **a** KRAS-driven tumors can produce several protumorigenic and immunosuppressive cytokines and factors, such as interleukins 1 beta (IL-1β), 6 (IL-6), 8 (IL-8), 10 (IL-10), 17 (IL-17), 35 (IL-35), transforming growth factor beta (TGF-β), granulocyte macrophage colony-stimulating factor (GM-CSF), colony-stimulating factor 1 (CSF1), vascular endothelial growth factor (VEGF), BCAM, metalloproteases (MMPs) and chemokines (C-X-C motif) ligands 1 (CXCL1), 2 (CXCL2), 3 (CXCL3), 5 (CXCL5) and 12 (CXCL12).Additionally, they can downregulate major histocompatibility complex class I (MHC-I) molecules. These mechanisms collectively lead to a reduced cytotoxic CD8+ T cell response and the recruitment of protumorigenic neutrophils, T helper 17 cells (Th17), myeloid-derived suppressor cells (MDSCs), M2 macrophages, regulatory T cells (Treg), regulatory B cells, endothelial cells, cancer-associated fibroblasts (CAFs), as well as to the extracellular matrix (ECM) remodeling. Furthermore, oncogenic KRAS induces overexpression of programmed death-ligand 1 (PD-L1) and cluster of differentiation 47 (CD47) on the membrane of the tumor cell, along with T cell exhaustion markers programmed cell death protein 1 (PD-1), cytotoxic T-lymphocyte-associated protein 4 (CTLA-4), and T-cell immunoglobulin and mucin-domain-containing-3 (TIM-3) on the surface of T cells. **b** Tumors with deregulated MYC produce immunosuppressive molecules like TGF-β, Twist-related protein 1 (TWIST1), lactic acid, chemokine (C-C motif) ligands 2 (CCL2), 5 (CCL5), 17, (CCL17), 22 (CCL22), IL-1β, IL-6, IL-10, IL-13, Aurora kinase A (AURKA), and inhibit MHC-I, interferon type I (IFN-I), CXCL13, STAT1, and STAT2. These molecules indirectly suppress cytotoxic CD8+ and helper CD4+ T cell responses, natural killer cells, dendritic cells and anti-tumoral B cells, while recruiting M2 macrophages, MDSCs, Treg, Th17, mast cells and endothelial cells. Additionally, these tumors can overexpress PD-L1, CD47 and CD276 on their surfaces. **c** In the case of tumors co-expressing oncogenic KRAS and deregulated MYC, there is increased expression of interleukin 23 (IL-23), chemokine (C-C motif) ligand 9 (CCL9), PD-L1 and CD47, as well as a reduction in IFN-I, CXCL13 and MHC-I. Ultimately, these molecules inhibit the cytotoxic CD8+ T cell response and the recruitment of T cells, natural killer cells, and anti-tumoral B cells while increasing immunosuppressive M2 macrophages and their vascular endothelial growth factor (VEGF)-derived angiogenesis. Created with BioRender.com

Beyond the impact that RAS and MYC cooperation has at a cell-autonomous level, it also significantly affects the immune system. In fact, these oncogenes act within the tumor immune microenvironment (TIME), contributing to immunosuppression and recruitment of immunosuppressive cells. This section explores the intricate mechanisms on how each oncogene, both independently and in collaboration, interacts with the immune microenvironment, fostering cancer immune evasion (Fig. 4).

### Oncogenic KRAS and TIME

Oncogenic mutations in KRAS significantly impact the TIME, steering it towards an immunosuppressed state that facilitates immune evasion. Below we explore the different mechanisms and immune subpopulations associated with such evasion in different cancer types (Fig. 4a).

#### Myeloid-derived suppressor cells (MDSCs)

Myeloid-derived suppressor cells (MDSCs), a heterogenous group of immunosuppressive myeloid cells, play a significant role in cancer progression by fostering metastasis and suppressing the immune system. The most frequent alteration derived from KRAS mutant-tumors is the elevated interleukin 6 (IL-6) secretion, induced by nuclear factor kappa-light-chain-enhancer of activated B cells (NF-kB). This cytokine leads to signal transducer and activation of transcription 3 (STAT3) signaling pathway activation, which ultimately triggers the accumulation of MDSCs in the TME of lung^215–217^ and pancreatic^218–220^ tumors. In this regard, pharmacological inhibition of IL-6 in KRAS-mutant lung cancer has shown to hinder tumor progression and reduce granulocytic MDSCs.^215^ Moreover, in KRAS-driven lung tumors, production of IL-6 together with transforming growth factor beta (TGF-β) has been demonstrated to induce the proliferation of T helper 17 (Th17) cells in the TME.^215,221^ These cells attract MSDCs by producing interleukin 17A (IL-17A).^222^ Eliminating Th17 cells (and the consequential absence of IL-17A) decreases tumor-cell proliferation and hinders MDSC recruitment.^223^ Furthermore, recent results revealed that mutant KRAS-G12D lung tumors exhibit high levels of IL-1β. Blockade of this interleukin reduces MDSC infiltration in the lung.^224^ In the case of CRC, KRAS mutations repress interferon regulatory factor 2 (IRF2), resulting in decreased IRF2/CXCL3 binding and, thus, higher CXCL3/CXCR2 coupling on MDSCs, promoting their migration to the TIME.^225,226^ In addition, it has been shown that GM-CSF upregulation by pancreatic and colorectal tumors enhances the infiltration of MDSCs to the TME,^227–231^ especially the expansion of immunosuppressive Gr1^+^CD11b^+^ myeloid cells,^227^ adding another element to the association between oncogenic KRAS and immune evasion.

#### M2-polarized macrophages

M2-polarized macrophages are known for their anti-inflammatory properties, linked to immunosuppression and often associated with a poor prognosis in response to IO. Several studies have shown increased levels of interleukin 10 (IL-10) and TGF-β in the serum and tumor tissue of KRAS-mutant pancreatic and lung cancer patients.^232–236^ Some authors also observed that mutations in KRAS increase the secretion of these two anti-inflammatory molecules in PDAC, NSCLC and CRC, which cause M2 macrophages recruitment to the tumor site.^237,238^ Moreover, in KRAS-mutant lung tumors, IL-6 inhibition reduces anti-inflammatory macrophages,^215^ suggesting that KRAS-induced IL-6 might also be responsible for the M2 macrophages recruitment. CSF1R blockade in a KRAS-G12D orthotopic model of PDAC abrogated M2 macrophage infiltration,^239^ highlighting the role of CSF1/CSF1R axis in sustaining the M2 macrophage-derived immunosuppressive environment.

#### Regulatory T (Treg) cells

Regulatory T cells (Tregs) are a controversial population in tumor immunity. While certain authors argue that Tregs have a negative predictive value for immune checkpoint inhibitors (ICIs) sensitivity, others suggest that responding patients exhibit increased proportions of these cells.^240^ In line with the already mentioned immunosuppressive MDSCs, elevated IL-6 secretion by KRAS-mutant lung tumors is crucial for Tregs recruitment and driving of a pro-tumorigenic Treg response.^215^ However, IL-6 is not the only cytokine that regulates these cells. Several preclinical studies have shown that mutant KRAS can induce the conversion of conventional anti-tumorigenic CD4^+^FoxP3^-^ T cells to pro-tumorigenic CD4^+^FoxP3^+^ Tregs through the secretion of IL-10 and TGF-β via the MEK–ERK–AP1 axis activation in NSCLC, PDAC and CRC models,^225,241,242^ highlighting the importance of KRAS signaling in producing an immunosuppressive TIME. Indeed, the role of TGF-β in inducing the differentiation to CD4^+^FoxP3^+^ Tregs in pancreatic cancer cells is also confirmed by the blockade of the molecule or its receptor.^243^ Also in pancreatic models, GM-CSF induces Treg expansion at early metastasis.^229^ Notably, eliminating Tregs in mutant KRAS transgenic mice results in fewer lung tumors, underscoring their role in lung tumorigenesis.^242^

#### T helper 17 (Th17) cells

T helper 17 (Th17) cells have already been linked with the immunosuppressive role of oncogenic KRAS, since mutant KRAS-driven IL-6 and TGF-β secretions induce proliferation of Th17 cells, attracting MDSCs to the tumor site.^215,221,222^ Functional ablation of these cells in KRAS mutant models of NSCLC and PDAC results in loss of IL-17A and the consequent reduction in tumor progression, angiogenesis and recruitment of MDSCs.^223,244^ The same study found increased levels of Th17 cells and IL-17A in the TIME of pancreatic intraepithelial neoplasia (PanIN) KRAS mouse models, promoting its initiation and progression.^223^ High levels of IL-17A in plasma samples of NSCLC and PDAC patients correlated with poor prognosis.^223,245,246^

#### Cytotoxic T cells

While KRAS-G12D lung cancer mouse models show expanded CD8^+^ cytotoxic T cells,^221^ they display reduced capacity to recognize and eliminate cancer cells, due to KRAS-induced downregulation of major histocompatibility complex (MHC) class I molecules, highlighting another immunomodulatory effect of oncogenic KRAS in the TIME.^216,247^ In the case of mutant KRAS pancreatic tumors, IL-10 and TGF-β production suppresses cytotoxic CD8^+^ T cell-mediated tumor killing.^238^ As already mentioned, GM-CSF secretion by KRAS-driven PDAC cells enhances the infiltration of MDSCs and Treg expansion, which ultimately provokes the suppression of CD8^+^ T cell proliferation.^227–230^ Mutant KRAS-induced IL-6 also elevates exhaustion markers on the T cells infiltrating the TME, namely programmed cell death protein 1 (PD-1), cytotoxic T-lymphocyte associated protein 4 (CTLA-4), and T-cell immunoglobulin and mucin-domain containing-3 (TIM-3), acting as immune checkpoints in lung cancer.^217^ In the case of colorectal cancer, the relation between mutant KRAS and PD-1 is regulated by IRF2. Mutations in KRAS repress IRF2, facilitating the appearance of resistance mechanisms to anti-PD-1 immunotherapy.^226^ Additionally, in KRAS mutant lung cancer, oncogenic KRAS enhances programmed-death ligand 1 (PD-L1) expression via sustained p-ERK activation^248–250^ and by stabilizing its mRNA through the negative regulation of AU-rich element-binding protein tristetrapolin (TTP). Restoring TTP activity lowers PD-L1 expression, boosting the anti-tumor immune response.^251^ STAT3, activated by heightened IL-6, drives this regulation, enhancing the interplay between PD-1 and PD-L1, facilitating immune evasion.^216^ The increase in PD-1/PD-L1 expression due to oncogenic KRAS activation was also observed in pancreatic cancer.^252^ However, in colorectal cancer, KRAS mutations are associated to low PD-L1 expression.^253,254^ PD-L1, though, is not the sole checkpoint affected by KRAS. CD47, a “don’t eat me” signal for macrophages, is often overexpressed on cancer cells too. In this case, mutant KRAS activates the IL-6-derived PI3K/STAT3 pathway, hindering CD47 inhibition and leading to its overexpression.^255,256^

#### Neutrophils

Neutrophils, while not intricately tied to immune evasion, play a pro-tumorigenic role.^255^ In lung cancer, oncogenic KRAS induces IL-8 overexpression,^257^ directly stimulating neutrophil infiltration and the formation of neutrophil extracellular traps (NETs).^258^ This phenomenon is also observed in colorectal cancer when oncogenic KRAS is activated.^259,260^ Moreover, in lung tumorigenesis, mutant KRAS-driven IL-17A secretion by Th17 cells further boosts IL-6 and G-CSF levels, enhancing tumor-associated neutrophil invasion.^261^ Finally, oncogenic KRAS in lung tumors increases the secretion of neutrophil chemokines such as Chemokine (C-X-C motif) ligands 1 (CXCL1), 2 (CXCL2) and 5 (CXCL5), establishing a clear link between KRAS and neutrophil recruitment.^262^

#### B cells

B cells exhibit a controversial function in the TIME, both as pro- and anti-tumorigenic immune cells. Examining the interplay between oncogenic KRAS and B cells in lung cancer reveals that KRAS-driven TGF-β secretion hampers the activation of normal B cells.^263^ A subset of B cells with pro-tumorigenic role, the regulatory B cells, are increased in lung cancer patients.^264^ This same subset is implicated in promoting the initiation and progression of PanINs through the secretion of interleukin 35 (IL-35).^265^ Furthermore, researchers have identified a population of antibody-producing B cells that not only facilitate tumor progression in PDAC but also induce M2 polarization of macrophages.^266^ Moreover, the B1β subset of B cells demonstrated a tumor-protective role in pancreatic tumorigenesis.^267^ These diverse phenotypes of B cells underscore their immunosuppressive function in KRAS mutant tumors.

#### Cancer-associated fibroblasts (CAFs)

Another type of cells within the TIME that plays a pivotal role in immune suppression is the cancer-associated fibroblasts (CAFs), also referred to as tumor-associated fibroblasts. These cells originate from normal fibroblasts due to the secretion of several pro-tumorigenic cytokines by cancer cells. Specifically, KRAS mutant cancer cells stimulate the secretion of IL-6 and TGF-β, well-known immunosuppressive molecules, thereby transforming normal fibroblasts into CAFs.^260^ Furthermore, KRAS-driven tumor cells express CXCR2 and GLI1, an effector molecule of the Hedgehog signaling pathway, which also interacts with CAFs to enhance their immunosuppressive function.^268–270^ Although the mechanisms by which CAFs exert their immunosuppressive function vary across tumor types, several pro-tumorigenic cytokines are commonly expressed by these cells, including IL-6,^270,271^ TGF-β,^272,273^ and CXCL12.^273,274^ The secretion of IL-6 by CAFs and subsequent activation of STAT3 contribute to reduced dendritic cell (DC) function, diminished T cell immune response,^275^ and upregulation of PD-L1 in neutrophils.^276^ Additionally, TGF-β, besides being a crucial inducer of CAF activation, inhibits DC migration and T cell priming,^277,278^ while also polarizing macrophages toward a more M2-like immunosuppressive phenotype through increased expression of CXCR4,^279^ CD163, CD206, and PD-1.^280^ The ligand CXCL12, also known as stromal cell-derived factor 1 (SDF-1), mediates the recruitment of various immune subpopulations to the tumor site. Some researchers argue that CXCL12-producing CAFs lead to T cell exclusion from the TIME,^281^ supported by evidence that inhibiting its receptor CXCR4 in T cells enhances the response to checkpoint blockade in a PDAC model.^282^ Moreover, CXCL12 recruits immunosuppressive MDSCs to the tumor site in breast^283^ and hepatocellular carcinoma models,^284^ as well as Tregs in breast, ovarian, pancreatic, and lung cancer models.^285,286^ Additionally, several authors have identified other cytokines released by CAFs in PDAC models that interact with diverse myeloid cells, including interleukin 33 (IL-33), CXCL1 and serum amyloids A1 (Saa1) and A3 (Saa3).^271^ Finally, growth factors such as VEGF produced by CAFs contribute to the inhibition of DC maturation.^287^ These findings collectively underscore the undeniable role of CAFs in tumor immune evasion and the establishment of an immunosuppressive TIME, besides their well-established role in promoting tumor growth, angiogenesis, tumor metabolism, and metastasis.

#### Endothelial cells

Endothelial cells, while not categorized as immune cells, play a crucial role in the TIME, contributing significantly to tumor immune evasion. Among the factors driving their activation, VEGF stands out as the most potent activator in KRAS-driven tumors,^288–290^ though it is not the only one. KRAS mutant tumors secrete IL-8, which enhances tumor inflammation and facilitates the recruitment of endothelial cells to the TME in several cancer models.^257,291–293^ These tumors also produce CXCL1 and CXCL5, which play roles in both the recruitment and proliferation of endothelial cells.^294^ Researchers have observed that mutant KRAS colorectal cancer cells exhibit increased expression of BCAM and induce upregulation of LAMA5 in endothelial cells. This interaction is crucial for the vascular adhesion of colorectal cancer cells and promotion of tumor progression.^295^

#### Noncellular components of the TIME

Apart from the immune and stromal cells mentioned above, the TIME also comprises noncellular components, such as the extracellular matrix (ECM). Under normal conditions, this structure supports and connects tissues, preserving their physiological functions.^296^ However, in tumor tissues, the ECM undergoes abnormal alterations that promote cell growth, transformation, and metastasis.^25^ The formation of this abnormal ECM is influenced not only by tumor cells but also by infiltrating immune cells within the TIME. KRAS-driven tumors increase the expression of different proteases, like the serine protease urokinase-type plasminogen activator (uPA) and different matrix metalloproteinases (MMP), leading to ECM remodeling.^212^ Similarly, in pancreatic tumors, mutant KRAS induces the expression of eIF5A, subsequently activating ROCK1 and ROCK2.^297^ This activation results in ECM remodeling, elimination of physical restraints, and facilitates tumor growth invasion.^298^ The alteration of the immune compartment by mutant KRAS also contributes to ECM remodeling and tissue invasion.^299^ Various subpopulations of macrophages and leukocytes express different matrix metalloproteinases such as MMP-2^300^ and MMP-7,^301^ and cathepsin B,^302,303^ which repress E-cadherin-mediated cell adhesion, thereby promoting tumor cell motility and invasion.^302,303^ Additionally, tumor infiltrating immune cells secrete TNFα, which activates downstream signaling cascades like NF-kB, leading to increased expression of various MMPs and subsequent tumor invasion.^304^ Macrophage-derived TNFα enhances WNT/beta-catenin signaling in gastric cancers by activating AKT signaling.^305^ Therefore, the immunosuppressive phenotype of the KRAS-driven TIME can directly and indirectly alter the ECM, facilitating tumor growth and invasion.

### MYC deregulation and TIME

Like KRAS mutation, MYC deregulation in cancer cells fosters an immunosuppressive TIME by both altering the protein expression profile within the deregulated tumor cells and attracting immunosuppressive cells to the tumor site.^16,116,306^ On one hand, deregulated MYC directly affects the process of immunoediting, causing increased expression of nuclear Aurora kinase A (AURKA), which, in turn, induces the expression of PD-L1,^307^ and upregulation of CD47^308^ on tumor cells, protecting them from the attack of T cells and macrophages. On the other, overexpression of MYC initiates tumor-induced immune suppression through the crosstalk between tumor cells and different immune cell subsets in the TIME through the production of specific cytokines and chemokines. Here, we explore the main mechanisms by which MYC drives this immune evasion (Fig. 4b).

#### MDSCs, M2 macrophages and Tregs

As abovementioned with oncogenic KRAS, deregulation of MYC increases IL-6 production and thus promotes the recruitment of immunosuppressive cells like MDSCs, M2-polarized macrophages, Th17 cells, and Tregs.^309^ Indeed, several authors provide evidence that MYC overexpression recruits MDSCs and immunosuppressive macrophages in several cancer types.^310–316^ In hepatocellular carcinoma, for example, MYC collaborates with Twist-related protein 1 (TWIST1) to regulate the secretion of chemokine (C-C motif) ligand 2 (CCL2) and interleukin 13 (IL-13), thereby fostering the recruitment and polarization of M2 macrophages.^316^ Moreover, MYC-driven metabolic reprogramming also plays an important role in the TIME of HCC, since lactic acid derived from tumor cells can activate nuclear factor erythroid 2-related factor 2 (NRF2) in macrophages, regulating the metabolism and polarization towards M2.^317,318^ Additionally, Tregs differentiation is influenced by the glycolytic environment created by MYC overactivation.^306,319,320^ Lactic acid produced by the tumor cells triggers the nuclear factor of activated T cell 1 (NFAT1) translocation into the nucleus of Tregs, enhancing PD-1 expression and promoting immune escape.^306,321^ Furthermore, MYC mediates the secretion of immunosuppressive chemokine (C-C motif) ligands 17 (CCL17) and 22 (CCL22), TGF-β and IL-10, attracting and activating Tregs.^213,322,323^

#### Cytotoxic T and Natural Killer (NK) cells

The regulation of anti-tumor cytotoxic cells, such as effector T and NK cells, plays a pivotal role in MYC-related immune evasion. When MYC is activated in cancer cells, it can decrease the expression of MHC-I molecules,^324–326^ affecting antigen presentation and the consequent stimulation of T and NK cells.^213,327^ Also, activated MYC suppresses T and NK cell-mediated immune surveillance by inducing CD276,^328,329^ secreting TGF-β that decreases T cell response^330,331^ and by transcriptionally repressing STAT1 and STAT2, along with the type I interferon pathway.^213,332^ Additionally, MYC is associated with DC development and therefore interferes with antigen presentation through MHC-I-independent pathways.^333^ MYC-driven overexpression of CD47 interferes in the crosstalk between tumor cells and DCs, enhancing immune escape.^306,308^

MYC deregulation modulates other immune subsets and stromal cells that participate in the tumor immune evasion. In the case of pancreatic tumors, MYC-driven IL-1β secretion increases the proliferation of endothelial cells.^323^ Immunosuppressive chemokine ligands CCL2 and CCL5 are also induced by MYC activation, recruiting mast cells to the tumor site.^334^ Moreover, MYC mediates the exclusion of antitumoral B cells by decreasing CXCL13 production.^332^ Thus, MYC deregulation significantly contributes to shaping the TME towards an immunosuppressive phenotype, eliciting clear immune evasion.

### Interplay between RAS and MYC in the TIME

Upon examining how KRAS mutations and MYC dysregulation tailor the TIME and impact different immune populations in several tumor types, it is apparent that they share several mechanisms. This commonality arises from their connection within the same pathways, jointly fostering an immunosuppressed tumor environment (Fig. 4c). Despite extensive research, many aspects of this interaction remain elusive.

In this context, Kortlever et al. shed some light on the relationship between RAS and MYC in the pathogenesis of NSCLC. Indeed, using a KRAS-driven lung adenocarcinoma mouse model wherein both KRAS-G12D and deregulated MYC expression can be conditionally activated, the researchers present compelling evidence demonstrating the collaboration between these two oncogenes to instruct an immunosuppressive TIME. Notably, moderate levels of MYC effectively transform dormant KRAS-driven tumors into aggressive, highly proliferative, and inflammatory adenocarcinomas. In this setting, hyperactivation of MYC triggers the release of IL-23 and CCL9. The former facilitates the efflux of CD3+ T cells, B cells, and NK cells, while CCL9 orchestrates the recruitment of immunosuppressive macrophages, also inducing PD-L1 expression.^30,213,216,335,336^ This leads to T cell exhaustion and expulsion, promoting angiogenesis via VEGF released by macrophages.^213^ The importance of IL-23 and CCL9 is eloquently demonstrated by their simultaneous blockade, leading to an efficient reversal of all MYC-induced stromal changes and substantial tumor shrinkage.^30,213^

Another recent investigation demonstrates that inhibiting KRAS-G12C in a mouse model of NSCLC restrains the downstream activity of MYC, boosting interferon signaling and reducing immunosuppressive cytokine production. This supports better infiltration and activation of CD8+ T cells and enhanced presentation of neoantigens by MHC molecules.^337^ Cooperative action of MYC and KRAS via interferon pathway was also observed by Muthalagu and colleagues in pancreatic models. Specifically, in mice, MYC and mutant KRAS-G12D promote PDAC progression by suppressing the type I interferon pathway, consequently hindering the CXCL13-mediated recruitment of antitumoral B and NK cells.^332^

The immune checkpoints PD-L1 and CD47 represent a crucial link between KRAS and MYC during immune evasion mechanisms. PD-L1 expression correlates with MYC levels in numerous cancer types.^213,338–341^ In NSCLC for example, the knockdown of MYC using small interfering RNA results in reduced PD-L1 mRNA and protein expression.^341^ On the other hand, as previously highlighted, oncogenic KRAS enhances PD-L1 by negatively regulating TTP, and the restoration of TTP reduces PD-L1 expression.^251^ Other authors also studied KRAS-mediated PD-L1 upregulation in lung^249,250^ and pancreatic^342^ cancers. Collectively, these findings highlight the relationship between KRAS mutation, MYC deregulation and PD-L1 overexpression, making them promising targets for lung cancer immunotherapy. In fact, several clinical trials combining KRAS inhibitors with PD-1/PD-L1 blockade are underway, although preliminary data have indicated only moderate success so far.^343,344^ In the case of CD47, its upregulation can be induced by both dysregulated MYC^306,308^ and activated KRAS.^255,256^ This underscores its significance as a target in immune evasion mechanisms within tumors featuring alterations in both oncogenes.^344^ Furthermore, although not traditionally classified as an immune checkpoint, MHC-I also plays a role in immune response and evasion. This receptor is downregulated in numerous MYC-driven tumors,^324–326^ resulting in diminished antigen presentation and reduced recruitment of T and NK cells.^213^ Also oncogenic KRAS diminishes MHC-I expression,^262,345^ suggesting a potential collaboration between the two oncogenes in fostering tumor immune evasion. Certainly, KRAS-targeted immunotherapy combinations are gaining traction in the clinical setting, particularly through the blockade of the PD-1/PD-L1 axis, although this exploration extends to other immune checkpoint inhibitors beyond PD-(L)1.^344^

Delving into the mechanisms underlying the cooperation between KRAS and MYC oncogenes and their impact on the immune microenvironment holds paramount importance for the development of novel and potent combinations in the field of immuno-oncology and patient selection.

## Therapeutic opportunities of targeting RAS and MYC in cancer

**Fig. 5:**
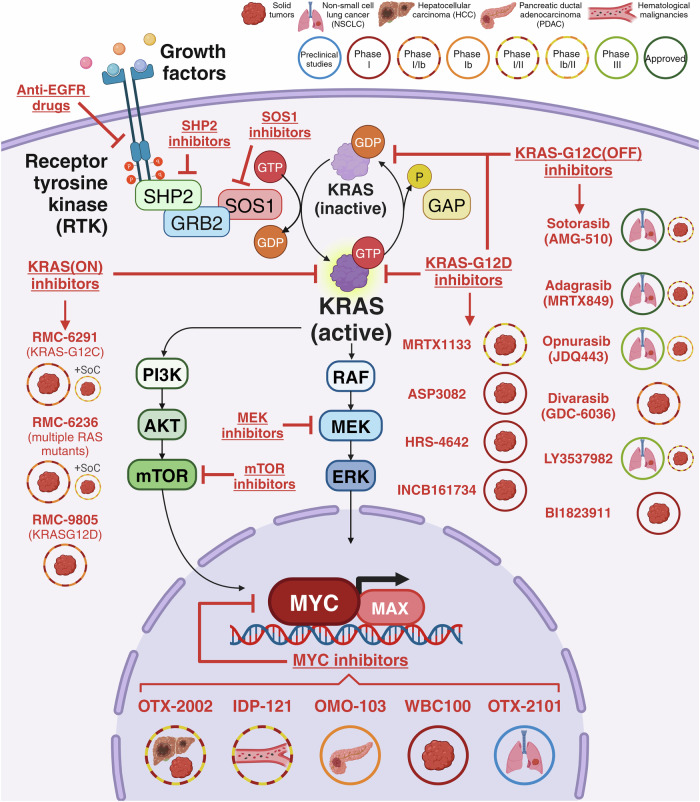
Clinical therapeutic strategies to target KRAS and MYC. Scheme of the main proteins involved in the KRAS signaling pathway, as well as several reviewed inhibitors, their clinical stage and the cancer type they are used in. The different inhibitor groups are underlined: molecules that inhibit the epidermal growth factor receptor (EGFR); Src homology region 2 domain-containing phosphatase-2 protein (SHP2) inhibitors; the Son of Seventless 1 protein (SOS1) inhibitors; the mammalian target or rapamycin molecule (mTOR) inhibitors, the MAP/ERK kinase (MEK) inhibitors; inhibitors of KRAS with the G12C mutation in its inactive state (KRAS-G12C(OFF)); inhibitors of KRAS with the G12D mutation in both its inactive and active state (KRAS-G12D); inhibitors of KRAS in its active state (KRAS(ON)); and MYC inhibitors. Among the KRAS-G12C(OFF) inhibitors, Sotorasib (AMG-510) and Adagrasib (MRTX849) are already approved for non-small cell lung cancer (NSCLC) and in Phase I/II for other solid tumors. Opnurasib (JDQ443) is in Phase III clinical trial for NSCLC and in Phase Ib/II for other solid tumors, while divarasib (GDC-6036) is in Phase Ia/Ib for all solid tumors. LY3537982 is in Phase III clinical trial for NSCLC and in Phase I/II for other solid tumors. Finally, BI1823911 is in Phase I clinical trial for all solid tumors. In the case of the KRAS-G12D inhibitors, MRTX1133 is in Phase I/II clinical trial for all solid tumors, while the rest of inhibitors, ASP3082, HRS-4642 and INCB161734, are in Phase I for all solid tumors. For what refers to the KRAS(ON) inhibitors, three promising small molecules are highlighted, RMC-6291, RMC-6236 and RMC-9805. The three of them are being evaluated as monotherapy in Phase I/Ib clinical trial for all solid tumors bearing G12C mutations, multiple RAS mutations and G12D mutations, respectively. Moreover, RMC-6291 and RMC-6236 are being tested in combination with the standard of care (SoC) in a Phase Ib/II clinical trial for all solid tumors. Four direct MYC inhibitors have reached clinical stage: OTX-2002 and IDP-121 are both in Phase I/II clinical trial, the first one for hepatocellular carcinoma (HCC) and other solid tumors and the second one for hematological malignancies; OMO-103 is currently in Phase Ib for metastatic pancreatic ductal adenocarcinoma (PDAC) patients in combination with chemotherapy, and WBC100 is in Phase I for solid tumors. Finally, OTX-2101 is under investigation in preclinical studies for NSCLC. Created with BioRender.com

Given the frequent occurrence of deregulations in both *MYC* and *RAS* in human cancers, these oncogenes emerge as highly appealing targets for cancer therapy. However, despite the preclinical demonstration that inhibiting MYC and RAS can be sufficient to induce sustained tumor regression,^213,214,346,347^ no drugs that directly target these oncogenes made it to the clinic until recently. In fact, their daunting complexity posed a remarkable challenge and historically classified them as “undruggable” entities for more than 40 years. Nevertheless, considerable progress has recently been made in the development of KRAS and MYC inhibitors for the treatment of cancer starting to show promising clinical results (Fig. 5), likely bringing the perception of these oncogenes as “undruggable” to an end.

### Therapeutic strategies to target KRAS

Direct therapeutic targeting of KRAS has been challenging for several reasons^348,349^: (i) KRAS protein surface appears notably smooth, featuring only one available GTP binding site; (ii) given the high concentration of GTP inside the cells and the exceptionally robust binding between GTP and KRAS, direct small-molecule inhibitors targeting GTP-binding pockets encounter substantial difficulty in competing with the binding; and (iii) KRAS is located inside the cell, a compartment not always easy to reach. For these reasons, the initial attempts to target KRAS were mainly focused on inhibiting membrane localization and upstream or downstream effectors. Following its folding process, KRAS experiences several post-translational modifications essential for its localization to the membrane (farnesylation, geranylgeranylation, and palmitoylation).^350^ Earlier endeavors aimed at inhibiting these enzymes, like farnesyl transferase inhibitors (FTIs), were a focal point in initial inhibition attempts but proved ineffective against solid tumors both as monotherapies and in combination with standard treatments.^350,351^

Several attempts have been made to inhibit KRAS activation by indirectly targeting its upstream effectors. Some drugs in this context are in clinical development but, to date, none of them have been approved for clinical use due to their modest therapeutic impact as single agents or even in combination. Both SHP2 and SOS1 inhibitors might hold some promise and have emerged as potential therapies targeting KRAS through disruption of its nucleotide exchange process. Inhibitors of these proteins that have reached clinical stage have been recently reviewed elsewhere.^352^ Another strategy is to inhibit signaling pathways upstream of KRAS, like EGFR. EGFR plays a vital role in cell proliferation and migration through the activation and signaling transduction of MAPK and PI3K.^353^ Currently, there are two types of anti-EGFR drugs available. One category comprises small-molecule TKIs, like gefitinib, which target the intracellular catalytic site of EGFR. The other category consists of monoclonal antibodies, like cetuximab, designed to bind to the extracellular domain of the receptor, thereby inhibiting receptor dimerization. EGFR inhibitors are employed in the treatment of various prevalent malignancies, such as breast, colon, lung, and pancreatic cancer.^353,354^ However, patients with advanced PDAC did not benefit from the addition of anti-EGFR antibodies to gemcitabine-backbone therapy.^355^

To target the downstream pathways of KRAS, numerous efforts have focused mainly on the MAPK and PI3K pathways, with somewhat disappointing results. While targeting downstream MAPK signaling proves effective for certain cancer types and stands as the standard treatment for patients with BRAF^V600E^ activating mutations,^356^ this approach has proved largely ineffective for KRAS mutant cancers. For instance, MEK inhibition using the small molecule trametinib or selumetinib showed initial promising results in early trials when combined with docetaxel but failed to improve survival of KRASmut NSCLC patients at later clinical stages.^357–359^ In the same line, early clinical trials of MEK inhibitors did not lead to satisfactory activity in PDAC.^355^ Selumetinib and trametinib had no significant effect on survival compared to capecitabine in gemcitabine-refractory PDAC patients,^360^ and the combination with gemcitabine was not associated with improved efficacy either.^361^ Several attempts have also been made to target the PI3K/mTOR/AKT pathway, unfortunately also yielding minimal success. For example, everolimus exhibited an overall response (OR) of merely 4.7% in a Phase II clinical trial involving advanced NSCLC patients,^362^ and no clinically relevant anti-tumor effects in PDAC patients as standalone treatment.^363,364^

Although inhibiting downstream effectors like the MAPK and PI3K pathways has shown success in some cancer types, it falls short in KRAS mutant patients. Targeting individual signaling nodes downstream of KRAS has not resulted in clinically meaningful responses, largely due to rapid adaptation and compensatory signaling through alternative pathways. Hence, joint inhibition of multiple key molecules seems to be required to achieve effective pathway suppression. Different strategies to indirectly target KRAS have thoroughly been reviewed elsewhere.^10,353,365^ In this review, we will focus mainly on direct mutant KRAS inhibitors which are already at the clinical stage. The most promising KRAS inhibitors undergoing clinical trials are listed in Table 1. Below, we summarize the current status of the most advanced ones.

**Table 1:** Direct KRAS inhibitors undergoing clinical trials

Inhibitor	Treatment strategy	Indication	Sponsor	Stage	Trial identifier
G12C inhibitors					
Sotorasib (AMG 510)	Monotherapy	Solid tumors	Amgen	Phase I/II	NCT03600883
		NSCLC	Amgen	Phase II	NCT04933695
		NSCLC	Vestre Viken Hospital Trust	Phase II	NCT05311709
		NSCLC	Fundación GECP	Phase II	NCT05398094
		NSCLC	Gustave Roussy	Phase II	NCT05631249
		Non-squamous NSCLC	SWOG Cancer Research Network	Phase II	NCT04625647
		NSCLC	Amgen	Phase III	NCT04303780
		NSCLC	Memorial Sloan Kettering Cancer Center	Phase II	NCT06333678
	Combination with stereotactic radiation therapy	Brain metastasis from NSCLC	Karolinska University Hospital	Phase I	NCT06127940
	Combination with SHP2 inhibitor	NSCLC; solid tumors	Navire Pharma Inc	Phase I	NCT05480865
	Combination with six other therapies	MTAP-deleted thoracic tumors	Amgen	Phase I	NCT06333951
	Combination with carfilzomib	NSCLC	City of Hope Medical Center	Phase I	NCT06249282
	Combination with tarloxotinib	NSCLC	Medical University of South Carolina	Phase Ib	NCT05313009
	Combination with ladaraxin	NSCLC	NYU Langone Health	Phase I/II	NCT05815173
	Combination with avutometinib	NSCLC	Verastem Inc	Phase I/II	NCT05074810
	Combination with DCC-3116	Solid tumors	Deciphera Pharmaceuticals LLC	Phase I/II	NCT04892017
	Combination with JAB-3312	Solid tumors; NSCLC	Jacobio Pharmaceuticals Co., Ltd	Phase I/II	NCT04720976
	Combination with zotatifin (eFT226)	Solid tumors	Effector Therapeutics	Phase I/II	NCT04092673
	Combination with 13 other anti-cancer therapies	Solid tumors	Amgen	Phase I/II	NCT04185883
	Combination with cisplatin (or carboplatin) and pemetrexed chemotherapy	Non-squamous NSCLC	M.D. Anderson Cancer Center	Phase II	NCT05118854
	Combination with RMC-4630	NSCLC	Revolution Medicines, Inc.	Phase II	NCT05054725
	Combination with panitumumab	Solid tumors	Korea University Anam Hospital	Phase II	NCT05993455
	Combination with panitumumab	Solid tumors	National Cancer Institute (NCI)	Phase II	NCT05638295
	Combination with 15 cancer therapies	Solid tumors	National Cancer Institute (NCI)	Phase II	NCT05564677
	Combination with platinum doublet chemotherapy (against pembrolizumab with platinum doublet chemotherapy)	NSCLC	Amgen	Phase III	NCT05920356
	Combination with panitumumab and FOLFIRI	CRC	Amgen	Phase III	NCT06252649
	Combination with panitumumab	CRC	Amgen	Phase III	NCT05198934
Adagrasib (MRTX849)	Monotherapy	Solid Tumors	Zai Lab (Shanghai) Co., Ltd	Phase I	NCT05263986
		PDAC	M.D. Anderson Cancer Center	Phase Ib	NCT05634525
		Solid Tumors	Mirati Therapeutics Inc.	Phase I/II	NCT03785249
		NSCLC	ETOP IBCSG Partners Foundation	Phase II	NCT05673187
		Solid tumors; malignant neoplasms of lung	Mirati Therapeutics Inc	Phase II	NCT05853575
		NSCLC	Mirati Therapeutics Inc.	Phase III	NCT04685135
	Combination with INCB099280	Solid tumors	Incyte Corporation	Phase I	NCT06039384
	Combination with KO-2806	Solid tumors	Kura Oncology, Inc.	Phase I	NCT06026410
	Combination with palbociclib	Solid tumors	Mirati Therapeutics Inc.	Phase I	NCT05178888
	Combination with cetuximab and irinotecan	CRC	M.D. Anderson Cancer Center	Phase I	NCT05722327
	Combination with olaparib	Solid tumors	M.D. Anderson Cancer Center	Phase Ib	NCT06130254
	Combination with nab-sirolimus	NSCLC; solid tumors	Mirati Therapeutics Inc.	Phase I/II	NCT05840510
	Combination with MRTX0902	Solid tumors	Mirati Therapeutics Inc.	Phase I/II	NCT05578092
	Combination with TNO155	Solid tumors	Mirati Therapeutics Inc.	Phase I/II	NCT04330664
	Combination with BMS-986466 with or without cetuximab	Solid Tumors	Bristol-Myers Squibb	Phase I/II	NCT06024174
	Combination with avutometinib	NSCLC	Verastem, Inc.	Phase I/II	NCT05375994
	Combination with SAR442720	Solid tumors	Sanofi	Phase I/II	NCT04418661
	Combination with pembrolizumab and cisplatin/carboplatin plus pemetrexed	NSCLC	Mirati Therapeutics Inc.	Phase II	NCT05609578
	Monotherapy and in combination with nivolumab	Resectable NSCLC	Sidney Kimmel Comprehensive Cancer Center at Johns Hopkins	Phase II	NCT05472623
	Combination with stereotactic radiation therapy	Brain metastasis from NSCLC	Ryan Gentzler, MD	Phase II	NCT06248606
	Monotherapy and in combination with pembrolizumab	NSCLC	Mirati Therapeutics Inc	Phase II/III	NCT04613596
	Combination with cetuximab	CRC	Mirati Therapeutics Inc.	Phase III	NCT04793958
Opnurasib (JDQ443)	Monotherapy	NSCLC	Novartis Pharmaceuticals	Phase II	NCT05445843
		NSCLC	Canadian Cancer Trials Group	Phase II	NCT05714891
		NSCLC with brain metastasis	Maastricht University Medical Center	Phase II	NCT05999357
		NSCLC	Novartis Pharmaceuticals	Phase III	NCT05132075
	Monotherapy and in combination with TNO155 and tislelizumab	Solid tumors	Novartis Pharmaceuticals	Phase Ib/II	NCT04699188
	Combination with trametinib, ribociclib and cetuximab	Solid tumors	Novartis Pharmaceuticals	Phase Ib/II	NCT05358249
Divarasib (GDC-6036)	Monotherapy	Solid tumors	Genentech, Inc.	Phase I	NCT04449874
		NSCLC	Genentech, Inc.	Phase II	NCT04302025
		NSCLC	Hoffmann-La Roche	Phase II/III	NCT03178552
	Combination with eight other anti-cancer treatments	CRC	Hoffmann-La Roche	Phase I	NCT04929223
	Combination with pembrolizumab, carboplatin, cisplatin and pemetrexed	NSCLC	Hoffmann-La Roche	Phase I/II	NCT05789082
	Combination with eight other anti-cancer treatments	Solid tumors	Hoffmann-La Roche	Phase II	NCT04589845
LY3537982	Monotherapy	Solid tumors	Eli Lilly and Company	Phase I	NCT04956640
		Solid tumors	Eli Lilly and Company	Phase I	NCT06235983
	In combination with pembrolizumab, cisplatin, carboplatin and pemetrexed	NSCLC	Eli Lilly and Company	Phase III	NCT06119581
BI-1823911	Monotherapy and in combination with BI-1701963	Solid tumors	Boehringer Ingelheim	Phase Ia/Ib	NCT04973163
RMC-6291	Monotherapy	Solid tumors	Revolution Medicines, Inc.	Phase I	NCT05462717
	Combination with RMC-6236	Solid tumors	Revolution Medicines, Inc.	Phase I	NCT06128551
	Combination with RMC-6236 or with SOC	Solid tumors with a focus on NSCLC	Revolution Medicines, Inc.	Phase I/II	NCT06162221
D-1553	Monotherapy	NSCLC	InventisBio Co., Ltd	Phase I/II	NCT05383898
		NSCLC	Chia Tai Tianqing Pharmaceutical Group Co., Ltd.	Phase III	NCT06300177
	Monotherapy and in combination with SoC (not determined)	Solid tumors	InventisBio Co., Ltd	Phase I/II	NCT04585035
	Combination with IN10018	Solid tumors	InventisBio Co., Ltd	Phase I/II	NCT05379946
	Combination with IN10018	Solid tumors	InxMed (Shanghai) Co., Ltd.	Phase I/II	NCT06166836
	In combination with SoC (not determined)	NSCLC	InventisBio Co., Ltd	Phase Ib/II	NCT05492045
JAB-21822	Monotherapy	Solid tumors	Jacobio Pharmaceuticals Co., Ltd.	Phase I/II	NCT05009329
		NSCLC	Jacobio Pharmaceuticals Co., Ltd.	Phase I/II	NCT05276726
		PDAC	Jacobio Pharmaceuticals Co., Ltd.	Phase II	NCT06008288
	Combination with JAB-3312	Solid tumors	Jacobio Pharmaceuticals Co., Ltd.	Phase I/II	NCT05288205
	Monotherapy and in combination with cetuximab	Solid tumors	Jacobio Pharmaceuticals Co., Ltd.	Phase I/II	NCT05194995
	Monotherapy and in combination with cetuximab	Solid tumors	Jacobio Pharmaceuticals Co., Ltd.	Phase I/II	NCT05002270
G12D inhibitors					
MRTX1133	Monotherapy	Solid tumors	Mirati Therapeutics Inc.	Phase I/II	NCT05737706
RMC-9805	Monotherapy	Solid tumors	Revolution Medicines, Inc.	Phase I	NCT06040541
ASP3082	Monotherapy and in combination with cetuximab	Solid tumors	Astellas Pharma Inc	Phase I	NCT05382559
HRS-4642	Monotherapy	Solid tumors	Jiangsu HengRui Medicine Co., Ltd.	Phase I	NCT05533463
INCB161734	Monotherapy and in combination with cetuximab or retifanlimab	Solid tumors	Incyte Corporation	Phase I	NCT05533463

#### G12C inhibitors

In 2013, seminal work conducted by Ostrem, Shokat and colleagues marked a significant milestone unveiling allele-specific covalent inhibitors targeting KRAS-G12C and paved the way for the discovery of a new generation of KRAS direct inhibitors. More in detail, the team identified an allosteric pocket specific to the GDP state positioned behind switch-II. Through iterative processes, they crafted a potent small molecule that could lock KRAS-G12C in the inactive GDP-bound state blocking oncogenic cell signaling.^366^ This breakthrough discovery led to the development of mutant-specific compounds, including AMG510 and MRTX849, that entered in clinical studies.^367,368^ A clinical breakthrough occurred in 2021 with the approval of Sotorasib (AMG-510) for the treatment of locally advanced or metastatic KRAS-G12C NSCLC patients who have progressed after standard therapies, becoming the first clinically available KRAS-targeted drug.^369,370^ Subsequently, Adagrasib (MRTX849) demonstrated superior clinical efficacy across a variety of solid tumors,^371^ leading to its approval in 2022.

#### Sotorasib (AMG-510)

As mentioned above, Sotorasib was the first specific inhibitor of KRAS-G12C to enter clinical trials. It is a highly specific and irreversible small molecule inhibitor based on the preclinical tool compound ARS-1620.^366,372^ It operates by binding to the allosteric pocket, known as the switch II pocket, locking KRAS in the inactive GDP-bound state.^373^ In preclinical studies, sotorasib treatment effectively suppressed the MAPK signaling pathway, resulting in sustained and complete regression of tumors. Additionally, it demonstrated potential in enhancing the efficacy of chemotherapy and targeted agents.^367^ Of note, in models with functional immune systems, sotorasib treatment triggered a pro-inflammatory TME, which led to sustained remissions when used alone or in combination with ICIs.^367^

The pivotal study highlighting the clinical efficacy of sotorasib was the CodeBreak 100 trial (NCT03600883).^369,370^ This phase I/II study aimed to assess the safety, pharmacokinetics, and efficacy of sotorasib in previously treated individuals with advanced solid tumors carrying the KRAS-G12C mutation. In the phase II portion of the trial, the activity of sotorasib was specifically evaluated in patients with previously treated KRAS-G12Cmut advanced NSCLC. The overall response rate (ORR) stood at 37.1%, the median PFS was 6.8 months, and the median overall survival (OS) reached 12.3 months.^370^ Based on these positive results, sotorasib was granted breakthrough designation by the U.S. Food and Drug Administration (FDA) in May 2021, specifically for treating adults with locally advanced or metastatic NSCLC with a KRAS-G12C mutation, who have progressed after at least 1 prior line of systemic therapy. To date, sotorasib has been approved in over 45 countries.^374^ These promising data led to an ongoing Phase III study (CodeBreak 200; NCT04303780) to evaluate the efficacy of sotorasib *vs*. docetaxel as second-line therapy in advanced KRAS-G12Cmut NSCLC. In this study, sotorasib significantly increased PFS (5.6 vs 4.5 months) and had a more favorable safety profile compared with docetaxel.^375^ Currently, sotorasib is being evaluated as first-line treatment in a Phase II trial (CodeBreaK 201; NCT04933695).

In patients with cancer types seldom presenting the G12C mutation, like PDAC and CRC, where these mutations represent less than 1.5% and 7% of all KRAS mutations, respectively, sotorasib has also shown some promising clinical activity. The objective response that was observed with sotorasib therapy in PDAC patients (21%)^32^ in the CodeBreak 100 trial was greater than that among patients with CRC (9.7%),^376^ but lower than the one observed for NSCLC (37.1%).^369,370^ Examination of data from CRC patients treated with KRAS G12C inhibitors indicates that, upon RAS inhibition, increased tyrosine kinase signaling within these tumors, coupled with the reactivation of receptors (particularly EGFR), diminishes the response to KRAS G12C inhibitors. Accordingly, single-agent activity of sotorasib can be improved by combination with the EGFR inhibitory antibody panitumumab.^377^

#### Adagrasib (MRTX849)

Adagrasib is another small-molecule inhibitor targeting KRAS-G12C, functions similarly to sotorasib by irreversibly binding to the mutant protein and effectively trapping it in the inactive GDP-bound state. It has been engineered for optimal pharmacokinetic properties, including high oral bioavailability, an extended half-life, broad tissues distribution, and the ability to penetrate the central nervous system.^378^ In preclinical evaluations, adagrasib demonstrated robust inhibition of KRAS-dependent signaling pathways and potent anti-tumor activity across various models derived from KRAS-G12C cell lines and PDXs.^368^

Results derived from the KRYSTAL-1 first-in-human Phase I/II clinical trial (NCT03785249) showed its safety and efficacy as monotherapy in previously treated KRAS-G12C NSCLC patients.^371,379^ At the recommended dose, the observed ORR stood at 42.9%, PFS was 6.5 months, and the median OS extended to 12.6 months.^371^ These positive findings substantiated the FDA’s accelerated approval of adagrasib in December 2022 for treating patients with locally advanced or metastatic NSCLC harboring KRAS-G12C mutations who have previously received at least one systemic therapy. Adagrasib is currently under comparison with docetaxel in a Phase III trial (NCT04685135; KRYSTAL-12) for previously treated KRAS-G12C NSCLC, and is also under investigation as a first-line treatment in a Phase II trial (NCT04613596; KRYSTAL-7).

Similar to sotorasib, adagrasib is currently under evaluation in a multiple expansion cohort of the KRYSTAL-1 phase I/II trial, including advanced solid tumors harboring a KRAS-G12C mutation. In the PDAC cohort, the ORR was 33.3% with a median PFS of 5.4 months.^380^ Previous data reported from other cohorts of this trial already demonstrated clinical activity and tolerability of adagrasib in previously treated patients with KRAS-G12C CRC. An objective response rate of 19% was reported, with a median duration of response of 4.3 months, and PFS of 5.6 months. However, in cohorts receiving adagrasib in combination with cetuximab, the objective response rate increased to 46%, with a median duration of response of 7.6 months, and a PFS of 6.9 months.^381,382^ The FDA granted breakthrough therapy designation for adagrasib in combination with cetuximab for the treatment of patients with KRAS-G12C CRC in December 2022.

Initial findings have also been reported for adagrasib in additional gastrointestinal (GI) malignancies, such as pancreatic, biliary tract, and appendiceal cancers, along with non-GI tumors like ovarian and endometrial cancers, showing encouraging clinical efficacy across these diverse tumor types.^379,380,383,384^

#### Other KRAS G12C inhibitors in clinical trials

In the quest to conquer the KRAS inhibition field, a myriad of other covalent inhibitors targeting mutant KRAS are currently advancing through clinical development (Table 1). Nonetheless, it is important to note that making cross-trial comparisons is challenging due to differences in patient selection. Hence, it is premature to know whether these initial findings can lead to a discernible advantage in the ongoing phase 3 trials. Here we summarize the clinical results of other leading clinical candidates.

JDQ443 (opnurasib) is a stable atropisomer with a unique binding mode to G12C mutant KRAS that has demonstrated potent and selective dose-dependent antitumor activity in vitro and in vivo in mouse xenograft models.^385^ JDQ443 is now in clinical development, with encouraging early phase data reported from an ongoing Phase Ib/II clinical trial (NCT04699188; KontRASt-01). This first-in-human trial is testing the safety and efficacy of JDQ443 alone or in combination with TNO155 (SHP2 inhibitor) and/or tislelizumab (anti-PD1 antibody) in patients with KRAS-G12C*-*mutated solid tumors, including NSCLC and CRC patients.^386^ At its recommended phase 2 dose (RP2D), JDQ443 showed an ORR of 41.7% in NSCLC patients.^387^ Now, a phase III trial is underway to assess the effectiveness and safety of JDQ443 as a standalone treatment versus docetaxel in patients with KRAS-G12C mutant NSCLC (NCT05132075; KontRASt-02). Additionally, JDQ443 will be evaluated as a single-agent as a first-line treatment for patients with advanced NSCLC whose tumors harbor a KRAS G12C mutation and PD-L1 expression <1% regardless of STK11 mutation status, or a PD-L1 expression ≥1% and STK11 co-mutation. (KontRASt-06; NCT0545843). To our knowledge, no data for other types of tumors have been reported so far.

GDC-6036 (divarasib) is being evaluated in a Phase Ia/Ib study as a single agent and in combination with other anti-cancer therapies in advanced solid tumors with KRAS-G12C mutation. In an ongoing Phase I trial evaluating this novel molecule as a single agent in pretreated advanced KRAS-G12C patients (NCT04449874), in the NSCLC cohort, divarasib displayed an ORR of 53.4%, including one complete response, and a median PFS of 13.1 months. Among the CRC patients, the OOR was 35.9% and the median PFS 6.9 months. Regarding PDAC patients, only 7 were enrolled in the trial, of which three displayed partial response and four showed stable disease, with a resulting ORR of 43%.^388^ Additionally, divarasib is currently being evaluated in this Phase Ib trial in combination with cetuximab in patients with KRAS G12C CRC. Preliminary results display an ORR of 62.5% and a median PFS of 8.1 months.^389^

LY3537982 is the successor compound of the initial drug LY3499446 (NCT04165031) following setbacks caused by unforeseen toxicities. This new entity has shown promising preclinical data alone and in combination with other targeted agents.^390^ A Phase Ia/Ib study of LY3537982 in patients with KRAS-G12C advanced solid tumors is ongoing to test the safety and efficacy of the new compound (NCT04956640). It demonstrated a favorable safety profile and showed the first signs of efficacy, with an ORR of 60% in NSCLC patients, 42% in PDAC, and only 10% in CRC.^391^ In one cohort of 11 CRC patients, the combination of LY3537982 with cetuximab demonstrated an ORR of 45%. Building on these initial findings, the SUNRAY-01 pivotal study (NCT06119581) is forecasted for early 2024 to evaluate the potential enhanced efficacy of incorporating LY3537982 into the Standard of Care (SoC) in participants with untreated advanced NSCLC.

In recent preclinical reports, BI-1823911 appears to be more powerful than sotorasib and adagrasib.^392,393^ BI-1823911 has displayed promising effects in preclinical models in combination with the pan-KRAS SOS1 inhibitor BI-1701963.^392^ Currently, BI-1823911 is undergoing a Phase I trial (NCT04973163) to assess its safety and effectiveness both as a monotherapy and in combination with BI-1701963 (SOS1 inhibitor) in different types of advanced cancers, including lung cancer, colorectal cancer, pancreatic cancer, and bile duct cancer. Although recruitment is still ongoing, preliminary data presented at ESMO 2023 suggested that is generally well tolerated and displays clinical activity in monotherapy.^394^ Of note, BI-1701963 was already tested in combination with adagrasib, but the trial was terminated due to toxicity issues (NCT04975256).^395^

#### G12D inhibitors

Given that G12D, G12V, and G12R KRAS mutations are more prevalent in KRAS-driven cancers like PDAC and CRC, there is a pressing need for effective therapies targeting these mutations. There are several drugs aimed at KRAS G12D in development, three of which are already at the clinical stage. G12D inhibitors were developed using alternative approaches compared to G12C, since KRAS G12D is characterized by the absence of a reactive cysteine residue near the switch II region, rendering the covalent blockade of the KRAS active center unfeasible for this allele.

Leveraging insights from the structure of Adagrasib, Mirati Therapeutics employed unconventional hydrogen bonding and ion pair binding strategies targeting the Asp12 residue of KRAS G12D, culminating in the development of MRTX1133.^396^ This non-covalent compound targets both the inactive and active forms of KRAS-G12D.^397^ At preclinical level, MRTX1133 displayed a significant therapeutic effect in several PDAC, CRC and NSCLC models, inducing significant regressions.^397,398^ MRTX1133 has demonstrated synergy with EGFR inhibition in xenograft models of pancreatic and colorectal adenocarcinomas, as well as in various human NSCLC, PDAC, and CRC cell lines, particularly when combined with AKT inhibitors but not with MEK/ERK inhibitors.^397^ A phase I/II clinical trial is currently ongoing evaluating MRTX1133 in patients with advanced solid tumors displaying the KRAS G12D mutation (NCT05737706).

Another approach to targeting KRAS-G12D has been to develop bifunctional PROTAC degraders. PROteolysis TArgeting Chimera (PROTAC) protein degraders leverage the cell’s intrinsic protein-degradation machinery, specifically the ubiquitin-proteasome system, to selectively target proteins for elimination. In this regard, Astellas is developing ASP3082, which binds KRAS-G12D to a E3 ligase to degrade the protein, and has recently entered clinical trials (NCT05382559). In this Phase I trial the investigators will test the safety and preliminary efficacy of the compound alone and in combination with cetuximab in patients with advanced PDAC, CRC, NSCLC or other solid tumors bearing the G12D mutation.

Other KRAS G12D-specific small-molecule inhibitors also in early phases of clinical development include HRS-4642 and INCB161734. HRS-4642 is a highly selective G12D inhibitor currently being evaluated in a Phase I clinical trial in China (NCT05533463), with preliminary results indicating an ORR of 33% in patients with NSCLC and CRC.^399^ INCB161734 is under development for the treatment of advanced and metastatic solid tumors with KRAS G12D mutation, in particular PDAC, CRC, and NSCLC (NCT06179160).

#### RAS(ON) inhibitors

While KRAS-G12Ci have displayed encouraging effects in patients carrying KRAS-G12C mutations, disease progression tends to occur due to the emergence of resistance mechanisms. To overcome some of the current KRASi liabilities, Revolution Medicines has created novel strong small molecules inspired on natural macrocycles to block different forms of mutant KRAS on its active state (ON), by sticking it to the molecular chaperone cyclophilin A (CYPA). In preclinical investigations, the resulting CYPA:drug:KRAS-G12C tri-complex has showcased these new inhibitors as a potential superior alternative to existing KRAS-G12Ci (OFF).^400^ Tri-complex inhibitors selectively targeting active KRAS-G12C (RMC-6291) or multiple RAS mutants, currently excluding G12C, (RMC-6236) are currently being evaluated in Phase I/Ib clinical trials (NCT05462717, NCT05379985, respectively). The preliminary results disclosed at ESMO and AACR-NCI-EORTC 2023 meetings showed that both drugs presented acceptable safety profiles and first signs of clinical activity. In the NSCLC cohort, the RMC-6291 G12Ci showed partial responses in 43% of the cases, with a disease control rate (DCR) of 100%. In the CRC cohort, partial response was observed in 40% of the patients, with a DCR of 80%.^401^ Following the same trend, the RMC-6236 multi-RAS inhibitor demonstrated preliminary evidence of clinical activity with an ORR of 38% and a DCR of 85% in the NSCLC cohort, and an ORR of 20% in PDAC patients and a DCR of 87%.^402^ At the AACR 2024 annual meeting, the authors reported that, consistent with preclinical observations, RMC-6236 showed clinical activity in solid tumors beyond NSCLC and PDAC, and in tumors harboring KRAS or NRAS mutations beyond G12X.^403^ Both compounds are currently being evaluated in a Phase Ib/II clinical trial in combination with SoC or with each other in RAS-mutated solid tumors, with a focus on NSCLC (NCT06162221).

Continuing the trajectory of these more advanced RAS(ON) compounds, Revolution Medicines initiated a Phase I/Ib clinical trial in September 2023 to assess the safety and preliminary clinical activity of their novel G12D(ON) inhibitor RMC-9805 in locally advanced or metastatic solid tumors bearing a KRAS G12D mutation (NCT06040541). Preclinical findings presented at the AACR 2023 annual meeting indicated that RMC-9805 monotherapy induced tumor regressions in the majority of preclinical PDAC and NSCLC cancer models harboring KRAS G12D. Moreover, RMC-9805 combination therapies prompted regressions in CRC models that were comparatively less responsive to monotherapy.^404^ Additional RAS(ON) inhibitors in the company’s pipeline include RMC-0708 (KRAS Q61H), which is currently in IND-enabling development, RMC-8839 (KRAS G13C), and additional compounds targeting other RAS variants.

### Therapeutic strategies to target MYC

Due to its undeniable role in driving and perpetuating tumorigenesis, MYC has been marked as a “most-wanted” target in cancer therapy. Yet, the absence of any clinically approved MYC inhibitor persists to date. Like KRAS, direct therapeutic targeting of MYC has been challenging for several reasons attributed to multiple facets of MYC biology^111,405^: (i) MYC is a transcription factor mainly localized within the cell nuclei, an elusive compartment to conventional drugs; (ii) As a monomer, MYC lacks a defined structure, classifying it as an intrinsically disordered protein, without either hydrophobic pockets or catalytic activity, posing challenges for standard small molecule inhibitors; (iii) The MYC family encompasses three distinct proteins, MYC, MYCN, and MYCL, which share overlapping functions in some contexts, making targeting all three paralogs needed to achieve complete MYC inhibition; and not least of all (iv) the pivotal role of MYC in normal cell proliferation and tissue regeneration has significantly contributed to its “undruggable” status, fostering concerns that its inhibition could potentially lead to severe adverse effects in normal proliferative tissues.

Despite these challenges, multiple strategies have emerged to target MYC at different levels, from its transcription to protein stability. Early-stage inhibitors or those that have been discontinued have been reviewed elsewhere^24,111,114,116,406–408^ (Whitfield & Soucek manuscript in revision). Briefly, some of these MYC inhibitors are aimed at blocking MYC transcription. One method to do so involves stabilizing G quadruplex (G4) motifs in the MYC promoter. Clinical trials with APTO-253, a G4-stabilizing agent, were discontinued due to lack of efficacy.^409^ Another approach is inhibiting bromodomain and extraterminal domain (BET) proteins, like BRD4, which regulate transcription by binding to chromatin. BRD4 inhibitors, such as JQ1, have shown promise in suppressing MYC and tumor growth, but their broad effects on other genes limit their clinical use due to toxicity.^111^ Alternative approaches aim to hinder MYC translation, such as antisense oligonucleotides or RNA interference using short/small hairpin (shRNA) or small interfering (siRNA) RNAs. Among these, DCR-MYC, a MYC-targeting siRNA, reached Phase I and II clinical trials. However, the trials were halted by the sponsor due to inadequate gene-silencing outcomes. Other efforts have been directed to preventing MYC/MAX protein interaction and binding to DNA through small molecules inhibitors. One example of those is MYCi975, which, in preclinical studies, induced MYC degradation, impaired MYC-mediated gene expression, and suppressed tumor growth.^410,411^ Furthermore, akin to approaches for other previously deemed “undruggable” proteins, degrader technologies such as PROTACS are also being explored for MYC. However, this approach faces the issue of the protein’s inherently short half-life and continuous production by cancer cells in response to its elimination. The most advanced anti-MYC strategies that are in clinical stage are summarized below and in Table 2.

**Table 2:** MYC inhibitors in clinical development

Inhibitor	Pharmaceutical company	Mechanism	Indication	Clinical Trial	Trial identifier
OMO-103	Peptomyc S.L.	MYC dominant negative	Advanced Solid Tumors	Phase I/IIa; Monotherapy	NCT04808362
			PDAC	Phase Ib; Combination with Gemcitabine/Nab-paclitaxel in PDAC	NCT06059001
IDP-121	IDP Pharma	c-MYC degradation	Hematologic malignancies	Phase I/II; Monotherapy	NCT05908409
WBC100	Zhejiang University	c-MYC degradation	Advanced Solid Tumors	Phase I; Monotherapy	NCT05100251
OTX-2002	OMEGA Therapeutics	MYC epigenomic modulation	HCC; Solid tumors	Phase I/II; Monotherapy and in combination with SoC in HCC	NCT05497453

#### OMO-103

OMO-103, a 91 amino acid mini-protein, is derived from the MYC mutant Omomyc, which is based on the bHLHLZ domain of the human c-MYC protein, carrying four specific amino acid mutations altering its dimerization properties.^412^ Briefly, Omomyc is able to efficiently form homodimers and to heterodimerize with both MYC and MAX. While Omomyc/Omomyc and Omomyc/MAX dimers bind to E-box sequences as inactive protein complexes hindering transcription of MYC target genes, Omomyc/MYC dimers are unable to bind DNA.^412^ Consequently, Omomyc functions as a dominant negative regulator of MYC’s transcriptional activity by both sequestering MYC away from the DNA and occupying its target genes with trans-repressing protein dimers. Dr. Soucek’s seminal work started with the use of Omomyc as a transgene both in vitro and in vivo, demonstrating the therapeutic potential of MYC inhibition for the first time. Then, multiple research groups validated Omomyc in several cancer models, where it halted tumor progression and even induced tumor eradication, independently of the tissue of origin or driving oncogenic lesions.^214,407,412–422^ These studies also underscored that MYC inhibition, contrary to prior belief, was well-tolerated by normal proliferative tissues that simply slowed down their proliferation rate.^421^

A crucial leap toward clinical translation occurred when Dr. Soucek group discovered that the purified Omomyc mini-protein itself possessed unexpected cell-penetrating capabilities, suggesting the feasibility of pharmacological MYC inhibition in vitro an in vivo in different models of NSCLC.^414^ This pharmacological tool was subsequently developed by Peptomyc S.L. in a first lead product, OMO-103, which entered First-in-Human (FIH) trials in 2021 for patients with advanced solid tumors (NCT04808362). This Phase I trial, completed at the end of 2022, demonstrated safety, promising signs of target engagement and drug activity in all-comers solid tumor patients.^423^ With this outcome, OMO-103 marks a groundbreaking achievement as the first direct MYC inhibitor demonstrating promising clinical results. Currently, OMO-103 is undergoing evaluation in a Phase Ib study, combined with SoC Gemcitabine/Nab-paclitaxel, as first-line treatment for metastatic PDAC patients (NCT06059001).

#### IDP-121

IDP-121 is a stapled peptide specifically designed to target the c-MYC protein by disrupting MYC/MAX binding and inducing MYC monomer degradation. It has shown promise across various liquid and solid tumor cell lines and preclinical animal models.^424^ Recently advancing into clinical stages, its safety and early efficacy are being evaluated in a Phase I/II trial for MYC-driven hematological malignancies (NCT05908409). According to the sponsor, the initial patient was treated in late October 2023. Presently, no further information regarding this compound is available to the authors’ knowledge.

#### WBC100

WBC100 is an orally available molecule glue designed to selectively degrade c-MYC that has shown encouraging preclinical data in different c-MYC overexpressing mouse models. It specifically targets the nuclear localization signal 1 (NLS1)–Basic–NLS2 region of c-MYC, identifying this region as a potential site for drug intervention. By utilizing the proteasome pathway, it induces the degradation of c-MYC protein.^425^ Presently, it is under investigation in a Phase I trial involving patients with advanced solid tumors expressing c-MYC (NCT05100251). As of now, no outcome data from the trial has been made available.

#### OTX-2002

OTX-2002 represents a groundbreaking approach in targeting MYC indirectly through epigenomic modulation. As an engineered and programmable mRNA therapeutic named Omega Epigenomic Controllers (OECs), it exerts its action on MYC gene expression pre-transcriptionally by establishing epigenetic marks at distinct structural and regulatory elements within the *MYC* insulated genomic domain. Encouraging preclinical data in hepatocellular carcinoma (HCC) models have showcased its potential anti-tumor activity, both as a standalone therapy and in combination with standard treatment approaches for HCC. Currently undergoing evaluation in a Phase I/II trial, OTX-2002 is being investigated as a monotherapy and in combination with standard treatments in patients with HCC and other solid tumor types linked to the *MYC* oncogene (NCT05497453). Early results reported by the sponsor, as per a press release, have displayed promising safety profiles and notable on-target epigenetic alterations in a subset of patients with HCC.^426^

Leveraging the success of OTX-2002, the company is now advancing OTX-2101, an OEC utilizing lung tissue-targeting nanoparticles, for the treatment of NSCLC. Preclinical evidence shared at the 2023 AACR-NCI-EORTC international conference demonstrated the potent downregulation of MYC in multiple NSCLC cell lines by OTX-2101. Moreover, it effectively reduced tumor growth both independently and in combination with ICI or EGFR-targeted therapies in murine xenograft models, further supporting its clinical prospects.^427^

## KRAS inhibitors therapeutic resistance: unveiling the interplay with MYC deregulation

Like other targeted therapies, the emergence of resistance to KRASi in the clinical setting poses a significant challenge. Although strides have been made with drugs like adagrasib and sotorasib, their clinical impact falls short compared to TKIs targeting other genetic drivers such as *EGFR*, *BRAF*, *ALK*, and *ROS1*. This underscores the need to understand resistance mechanisms and develop effective strategies for KRASi to maximize their clinical efficacy. Growing evidence indicate that mechanisms of primary and acquired resistance to KRAS-G12C inhibition are heterogeneous and diverse, examples of which are too numerous to comprehensively cover here but have been mostly reviewed elsewhere.^351,428–432^ Briefly, primary resistance mechanisms include loss of function mutations in tumor suppressor genes, intratumoral heterogeneity with some cells having a lower degree of KRAS dependency, upregulation of wild-type RAS isoforms, adaptive feedback reactivation of upstream RTK signaling, etc. The mechanisms leading to acquired resistance against G12Ci are multifaceted, encompassing both on-target and off-target pathways. On-target resistance involves acquired secondary KRAS mutations (at codon 12, 13 and 61), and alterations within drug binding sites. Off-target resistance may arise from mutations in other members of the RTK-RAS-MAPK pathways, compensatory activation of RTKs as bypass survival signaling, histologic transformation from adenocarcinoma to squamous cell carcinoma, and oncogenic gene rearrangements and amplifications, among others. In this regard, up to 90% patients treated with divarasib +/− cetuximab exhibited acquired genomic alterations linked to treatment resistance. The most prevalent mechanisms of resistance observed included genomic KRAS alterations, together with alterations in components of the PI3K and RTK pathways, such as EGFR, MYC, and *MET* amplifications, as well as *ALK* and *RET* fusions.^388,389^

To address these bypass resistance mechanisms and improve treatment outcomes, combination approaches that target the complex RAS pathway are being explored. Several clinical trials combining KRASi with inhibitors of upstream (i.e. SHP2 and SOS1) and downstream (i.e. MEK, ERK, mTOR, PI3K, AKT) RAS effectors, as well as with RTK inhibitors like EGFR or even with ICIs are underway (Table 1).^351,374^ In addition to intensifying inhibition of the RAS signaling network, researchers have explored combinations that target downstream events within tumor cells. These include inhibitors of cell cycle-dependent kinases, such as the CDK4/6 drug palbociclib,^368^ mTOR kinase,^368,433,434^ and eIF4A,^435^ an RNA helicase crucial for cap-dependent translation.

Understanding resistance mechanisms in KRAS inhibitor treatment is pivotal in shaping effective therapeutic strategies. MYC, as mentioned above, often exhibits co-dependence and functional overlap with KRAS signaling pathways. It has already been described that MYC overexpression or dysregulation is associated with resistance to numerous cancer therapies, including chemotherapy, targeted therapy, and immunotherapy. MYC activation contributes to many cancer hallmarks, including proliferation, self-renewal, cell survival, and genomic instability, all of which are associated with therapeutic resistance. Additionally, MYC alters the TME creating conditions that hinder drug delivery and immune responses, fostering therapeutic resistance. Understanding the interplay between MYC dysregulation and KRASi resistance offers insights into co-targeting strategies to potentially enhance treatment efficacy in KRAS-driven cancers. In this regard, several preclinical evidence points to this direction. One study revealed that KRAS-dependent suppression of MYC enhances the sensitivity of cancer cells to cytotoxic agents, indicating that MYC suppression is mediated by increased and prolonged activation of the MAPK/ERK pathway.^31^ Additionally, it was found that ERK inhibitor-resistant PDAC possesses ERK-independent mechanisms that maintain MYC protein stability, antagonizing KRAS suppression-induced degradation of MYC.^156^ Furthermore, targeting ST8SIA6-AS1 was shown to counteract KRAS-G12Ci resistance by abolishing the reciprocal activation of PLK1/MYC signaling, providing further insight into the role of MYC in resistance to KRAS inhibitors.^436^ Similarly, targeting CDK9 in KRAS-mutant PDAC cell lines resulted in MYC loss through both transcriptional and posttranslational mechanisms, effectively suppressing PDAC growth. This study illustrates that CDK9 enhances MYC protein stability by directly phosphorylating MYC at Ser62.^437^ Recently, Macaya et al. elegantly demonstrated that MYC upregulation contributes to both MEKi and KRASi resistance in NSCLC, as MYC levels and its transcriptional activation were increased in resistant cells compared to sensitive ones. In addition, exogenous MYC overexpression enhanced resistance to both therapies, indicating a functional link between MYC and the resistant phenotype.^438^ In light of these findings, clinical trials exploring direct MYC inhibitors in combination with KRASi may pave the way for evaluating enhanced therapeutic responses in KRAS-driven tumors.

## Conclusions and perspectives

Historically labeled as “undruggable”, both KRAS and MYC have posed significant challenges in therapeutic targeting due to their complexity. However, recent advancements in direct KRAS-G12Ci and the introduction of the first direct MYC inhibitors into the clinic mark a significant turning point in cancer therapeutics. Recently, breakthroughs in targeting KRAS and MYC have been achieved, with promising emerging clinical results, signifying the end of their “undruggable” status. The journey towards effective KRAS inhibition has faced substantial challenges, but the recent development of direct KRAS-G12Cis, notably sotorasib and adagrasib, has transformed the landscape of KRAS-targeted therapy, culminating in their clinical approval and paving the way for novel and better performing drugs. Similarly, the pursuit of MYC inhibition has encountered obstacles attributed to the multifaceted nature of MYC biology, but therapeutic strategies targeting MYC have made significant strides, with promising candidates advancing into clinical stages. In this context, the promising clinical results of OMO-103 represent a game-changing achievement.

However, even if both KRAS and MYC have important roles in tumor progression individually, their cooperative interactions significantly drive cancer initiation, progression, and therapeutic resistance. Further investigation into these interactions will provide valuable insights into developing novel co-targeting strategies to improve treatment outcomes in poor prognosis cancers like PDAC, advanced CRC and NSCLC. Emerging evidence has highlighted the interplay between MYC dysregulation and resistance to KRAS inhibitors, emphasizing the potential for synergistic effects of combined MYC and KRAS inhibition strategies. The importance of demonstrating this synergistic combination in preclinical models is an essential step towards informing clinical trial design and maximizing the likelihood of success. Considering the cooperative role of both MYC and KRAS in shaping the anti-tumor immune response, prioritizing preclinical experiments with immune-competent models, such as genetically engineered mouse models and syngeneic tumor models, becomes essential. Future research efforts should continue to leverage these preclinical models to optimize therapeutic strategies and accelerate the translation of findings into clinical trials.

Like for any other combinatorial approach, combining drugs targeting MYC and KRAS presents several challenges. Firstly, potential synergistic toxicity must be carefully managed. In fact, valuable information is already emerging from studies combining KRAS inhibitors with other compounds.^10,344,439,440^ Whether this same toxicity will manifest with direct MYC inhibitors or it could be exacerbated by them remains to be seen. As for other therapies, potential pharmacokinetic interactions between the drugs may alter their bioavailability and efficacy, thus necessitating optimized dosing regimens.

**Fig. 6:**
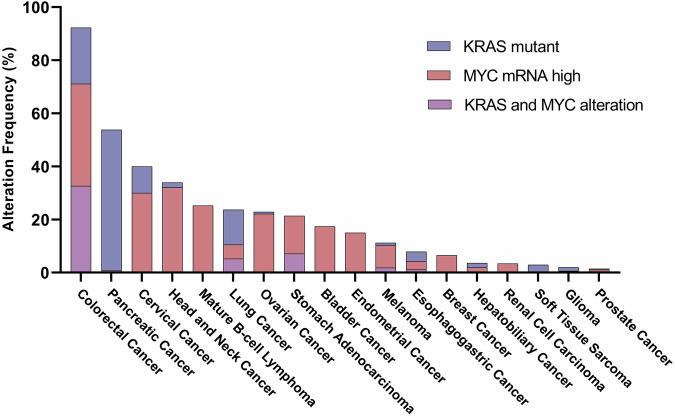
Frequency of KRAS mutations and high *MYC* mRNA expression in different tumor types. Percentage of cases presenting mutations in KRAS are indicated in purple, high *MYC* mRNA expression (standard deviation >1) in red, and co-occurring alterations in pink. Data acquired from cBioPortal using the Pan-Cancer analysis of whole genomes (ICGC/TCGA) dataset, which includes 2658 whole-cancer genomes across 38 tumor types from the International Cancer Genome Consortium (ICGC) and The Cancer Genome Atlas (TCGA)(ICGC/TCGA Pan-Cancer Analysis of Whole Genomes Consortium. Pan-cancer analysis of whole genomes. Nature. 2020). Only tumor types with more than 25 samples and 1% of altered cases were included in the analysis

Importantly, patient stratification based on molecular profiles and predictive biomarkers may be crucial for identifying individuals most likely to benefit from this combination treatment. An ideal scenario would involve cancer patients exhibiting both KRAS mutations and MYC deregulation (Fig. 6). In this context, while KRAS mutations may serve both as predictive and prognostic biomarkers, MYC has yet to be defined as a biomarker within a clinical setting.^441^ It is important to note, for example, that *MYC* amplification or high *MYC* mRNA expression does not always correlate with elevated MYC protein levels and, even less, with MYC activity.^423^ In fact, MYC addiction is not necessarily dependent on it absolute levels, but more on its tonic, deregulated signal.^146^ Therefore, including MYC amplification/overexpression as an inclusion criterion for clinical trials investigating combination of KRAS and MYC inhibition may be misleading and requires careful consideration. Clinical trial design must account for these complexities, ensuring robust endpoints, valid patient selection criteria, and rigorous regulatory approval pathways. Looking ahead, clinical trials evaluating direct MYC inhibitors together with KRAS inhibitors offer a promising direction potentially reshaping the treatment landscape for KRAS-driven cancers.
